# Multiple pathways towards achieving a living income for different types of smallholder tree-crop commodity farmers

**DOI:** 10.1007/s12571-021-01220-5

**Published:** 2021-10-18

**Authors:** Y. R. Waarts, V. Janssen, R. Aryeetey, D. Onduru, D. Heriyanto, S. Tin Aprillya, A. N’Guessan, L. Courbois, D. Bakker, V. J. Ingram

**Affiliations:** 1 Wageningen Economic Research, Wageningen University and Research (WUR), P.O. Box 29703, 2502 LS The Hague, The Netherlands; 2 Forest & Nature Conservation Policy Group, Wageningen University and Research (WUR), Wageningen, The Netherlands; 3 School of Public Health, University of Ghana, Legon, Ghana; 4 ETC Consultants, Nairobi, Kenya; 5 CIRCLE Indonesia, Yogyakarta, Indonesia; 6 EMC - Etudes de Marche Et Conseils, Abidjan, Ivory Coast; 7 Imani Development, Blantyre, Malawi

**Keywords:** Smallholder commodity farmers, Poverty benchmarks, Living income, Behavioural change, Land governance, Social assistance programme

## Abstract

Many sources indicate that smallholder tree-crop commodity farmers are poor, but there is a paucity of data on how many of them are poor and the depth of poverty. The living income concept establishes the net annual income required for a household in a place to afford a decent standard of living. Based on datasets on smallholder cocoa and tea farmers in Ghana, Ivory Coast and Kenya and literature, we conclude that a large proportion of such farmers do not have the potential to earn a living income based on their current situation. Because these farmers typically cultivate small farm sizes and have low capacity to invest and to diversify, there are no silver bullets to move them out of poverty. We present an assessment approach that results in insights into which interventions can be effective in improving the livelihoods of different types of farmers. While it is morally imperative that all households living in poverty are supported to earn a living income, the assessment approach and literature indicate that focussing on short- to medium-term interventions for households with a low likelihood of generating a living income could be: improving food security and health, finding off-farm and alternative employment, and social assistance programmes. In the long term, land governance policies could address land fragmentation and secure rights. Achieving living incomes based on smallholder commodity production requires more discussion and engagement with farmers and their household members and within their communities, coordination between all involved stakeholders, sharing lessons learnt and data.

## The living income concept in the context of smallholder tree-crop commodity production

### Smallholder tree-crop commodity production, poverty and intervention impacts

#### Millions of smallholder tree-crop commodity farmers produce the raw material for tea, coffee, chocolate and other products, and many of them are poor

Millions of people globally, including many smallholder farmers, earn a revenue from the cultivation or processing of agricultural commodities, particularly tree-crops such as cocoa, coffee, cotton, oil palm and tea (Voora et al., 2019a, b, c, d, 2020a, b) (Table 1). Tree crops, given their long maturation and system lifecycles, have particular distinguishing farming and farmer system characteristics (Ingram, 2021). Commodity production and processing are thus important economic activities within local food systems. The production of these crops largely takes place in lower- and middle-income countries; throughout the literature, it is clear that many of the smallholder farmers in these commodity sectors are poor (Voora et al., 2019a, b, c, d, 2020a, b). They have no control over global market prices and are often hampered by limited negotiating power. They are vulnerable to price changes in markets as well as climate change (International Food Policy Research Institute (IFPRI), 2020). In times of oversupply and market speculation, commodity prices can fall below the cost of production so that smallholder farmers cannot break even. Prolonged periods of low prices can have a disastrous effect on farmers' livelihoods and on the long-term sustainability of commodity supply. Poverty adversely affects human wellbeing and development, including productivity, and overcoming it remains a central focus of global sustainable development goals (United Nations, 2020).

**Table 1:** Overview of the number of people earning a revenue from commodity production and processing

**Sector**	**Number of people earning revenue from cultivation and processing**	**Total number of farming households**	**Share of smallholder farmers in total number of farming households**
Cocoa (Voora et al., 2019a)	40–50 million	5 million	70% (3.5 million)^*^
Coffee (Voora et al., 2019b)	125 million	12.5 million	67–80% (8.4–10 million)^*^
Oil palm (Voora et al., 2019d)	About 6 million		3 million smallholders
Tea (Voora et al., 2019c)	Over 13 million		9 million smallholders 70% of global production comes from 8 million smallholder farmers in Asia and Africa

^*^Author’s calculations based on (Voora et al., 2019a, b, c, d)

#### To date, the success of interventions aimed at reducing poverty levels of smallholder commodity farmers has been limited

Many different types of interventions have been implemented in tree-crop commodity value chains in the past two decades, by private and public sector, and non-governmental organisations. Most interventions have focused on improving productivity or enhancing local capacities or structures (Ingram et al., 2018). Examples of such interventions are training on agricultural practices, voluntary sustainability certification, provision of free or subsidised inputs such as seeds and fertiliser, support to farmer groups, community-level provision of infrastructure, and access to finance. But such interventions generally have either not lifted smallholder farmers out of poverty, or their effectiveness has not been documented, as most interventions that have been documented have had limited, mixed or no impact on household incomes (Alvarez & Von Hagen, 2011; Dalberg & Wageningen University, 2018; Ingram et al., 2014, 2018; Oya et al., 2017; Waarts et al., 2015, 2016; Woodhill et al., 2020).

### The living income concept and how it is used

#### The concept of living income embraces ‘a decent standard of living’ for households

**Fig. 1:**
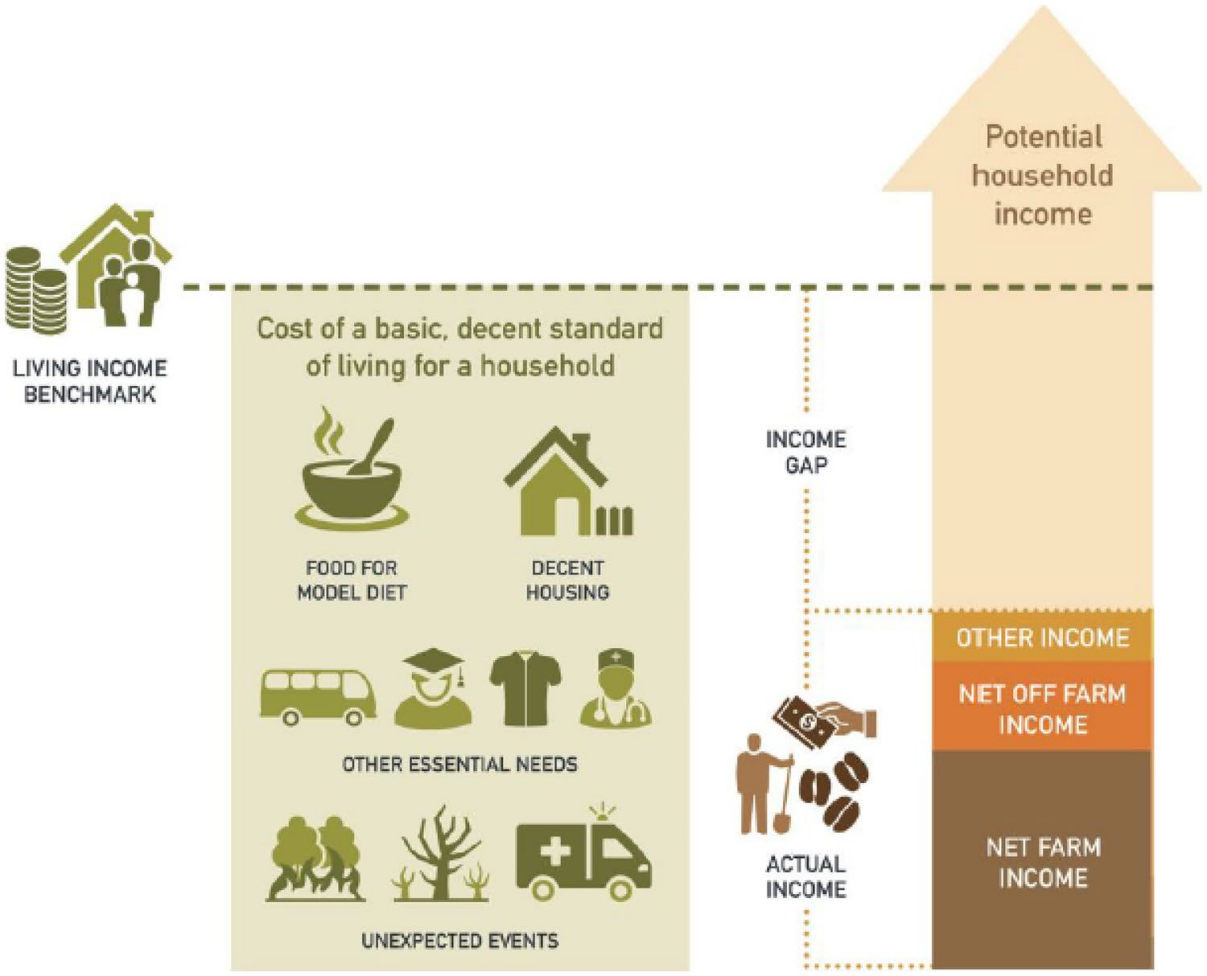
The living income concept. Source: Living Income Community of Practice: www.living-income.com

World Bank poverty lines are commonly used to assess poverty levels and compare countries, especially, the extreme poverty line of USD 1.90 (2011 PPP). But stakeholder groups increasingly realise that such poverty lines are not an indication of whether farmers have a decent standard of living. Instead, the aim of poverty measurements should be to focus on empowering people to have a ‘decent standard of living’ (Minos, 2018). This is connected to clause 25 of the Universal Declaration of Human Rights that states that: ‘Everyone has the right to a standard of living adequate for the health and well-being of himself and of his family, including food, clothing, housing and medical care and necessary social services, and the right to security in the event of unemployment, sickness, disability, widowhood, old age or other lack of livelihood in circumstances beyond his control’. Due to the growing interest of donors, NGOs, policy makers, and other parties to achieve a decent standard of living, the World Bank poverty benchmarks are gradually being replaced with ‘living income’ and ‘living wage’ benchmarks in commodity sectors, to assess poverty levels and impact of interventions on poverty. A living income is defined as ‘the net annual required for a household in a particular place to afford a decent standard of living for all members of that household’ (Anker & Anker, 2017) (Fig. 1). A ‘decent standard of living’ includes: a nutritious low-cost diet based on nutritional requirements and local food preferences, housing that meets local norms and common international standards of decency, essential needs including healthcare, clothing, education and transport, and a margin for unforeseen events (Anker & Anker, 2017; Grillo, 2018). The margin for unforeseen events anticipates and plans for resilient livelihoods. Whereas living wages focus more on wages from single sources (e.g. factory workers), the living income concept considers all sources of income for the entire household.

#### To assess the extent farmers earn a living income, information on actual total net household income levels is needed, and a living income benchmark needs to be established

To know what a living income is, a living income benchmark is established for a specific country, or region within a country. The circumstances within a particular year or season, cost of living, price changes over time, and inflation are considered. A living income benchmark thus indicates what a typical household minimally needs to have ‘a decent life’, based on the cost of a basic, decent standard of living for that household. Such benchmarks are often linked to a specific sector and/or a certain location. To establish this benchmark, information is collected from national and regional statistics and/or field research is conducted to obtain information (CIRES, 2018; van de Ven et al., 2020). To calculate the gap between actual incomes and a living income, the actual net household income per household member per day is deducted from the living income benchmark per household member per day (COSA and KIT, n.d.; Impact Institute, n.d.)^1^. The formula to calculate the gap between the living income benchmark and actual household income is shown in Formula 1. It should be mentioned that even if the living income concept is an entire household concept, it may be that even if a household earns a living income, such income and benefits stemming from the income, are not divided equally between all household members. This should be addressed in conducting living income assessments.

**Formula 1** The formula for calculating the living income gap

$$LI:Living\;income\;benchmark\;per\;person\;per\;year=\frac{Living\;income\;benchmark\;per\;month\;for\;a\;typical\;household\ast12}{Number\;of\;typical\;household\;members}$$

$$AI:Actual\;total\;net\;household\;income\;per\;person\;per\;year=\frac{Actual\;total\;net\;income\;per\;household\;per\;year}{Number\;of\;household\;members}$$

$$Living\;income\;gap\;per\;person\;per\;day:\frac{LI-AI}{365}$$

### The food systems approach

#### In the food system, income is interlinked, often nonlinearly, with food and nutrition security

**Fig. 2:**
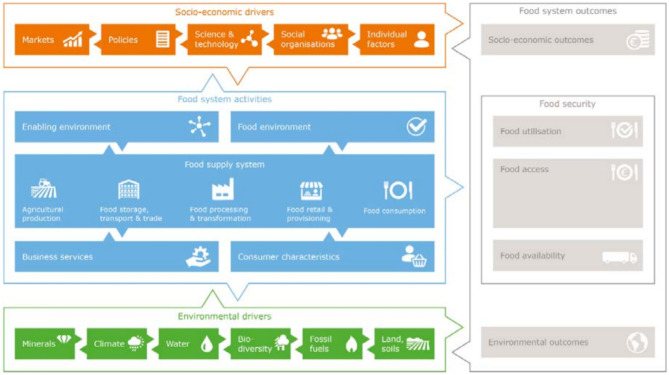
The food system, its drivers and outcomes. Source: (van Berkum et al., 2018)

Food and nutrition are both influenced by and influence health, labour availability, household decision-making, yields and incomes (Walton et al., 2020) as well as farm productivity (Arsyad et al., 2019; Fisher & Hostland, 2002). A food systems approach, defined as all the processes involved in achieving desired food system outcomes, including but not limited to food security for a specific population (Fig. 2), is the framing concept used to examine living incomes and poverty levels. This is due to the strong effect of household incomes on food security outcomes (Babatunde & Qaim, 2010; Iram & Butt, 2004; Kennedy & Peters, 1992). Poverty status and the living income concept are used as a lens to focus on smallholder tree-crop commodity farmer household incomes as socio-economic food system outcomes.

#### In a food system, contextual and personal factors influence farmer’s decision making and behaviour

**Fig. 3:**
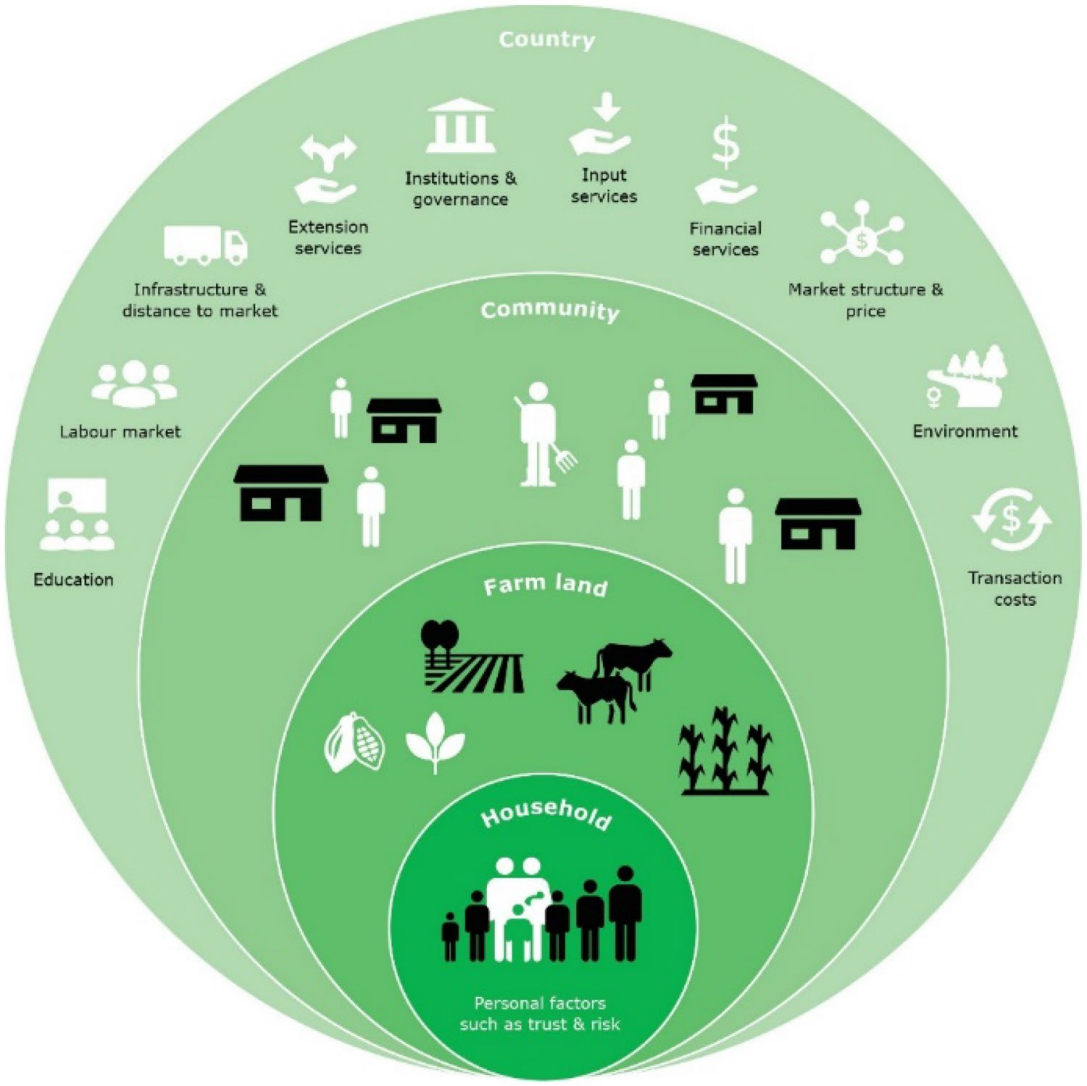
Key contextual factors in the design of living income interventions. Source: (Waarts et al., 2019)

Within a food system, contextual factors (shown in Fig. 3) and personal factors, can strongly differ between farmers and geographies (van Berkum et al., 2018; Waarts et al., 2019). These factors have important implications for farmers’ income from agricultural production and off-farm activities, which is why we present the two figures additionally to the food systems figure above as it deepens the information for food system components which are important for tree-crop commodity farmer income. Regarding contextual factors, what works best in one place, might not work in another place. Some of the key recurring barriers to behavioural change are: 1) Farmers might not be able to afford the financial investments that are required for technological innovations, or might decide to invest in something else, 2) The future benefits of investments are not guaranteed, 3) Failing markets lead to adoption constraints (i.e. shortage of inputs when needed), 4) Interventions may not be tailored enough to farmers’ specific needs and possibilities (Waarts et al., 2019). Interventions that address these barriers are therefore more likely to lead to behavioural change of farmers and therefore positively impact incomes.

#### Personal factors also influence whether farmers will adopt certain technologies or participate in interventions

Frequently, interventions have been implemented from a technocratic perspective which does not consider personal factors. Studies have shown that these factors influence farmer and household decision-making processes (Waarts et al., 2019). Personal factors include socio-economic characteristics and farmers’ aspirations, the effects of poverty on decision making and the cultural environment. Cultural factors can prevent or enable the uptake of certain interventions. Personal factors, like peer effects, are relevant for interventions, as often assumptions are made about diffusion of the results and outcomes of interventions to neighbours who did not participate in the intervention. It is important for interventions to make explicit such theories of change. In Farmer Field Schools, for example, assumptions about knowledge diffusion have not occurred as anticipated (Waddington et al., 2014).

### Objective of this paper

#### A living income and food systems approach are used to assess the potential of different types of interventions for smallholder commodity farmers to earn a living income

This paper sets out to show how the extent to which smallholder commodity farmers are poor and the depth of poverty, for cocoa and tea farmers in Ghana, Ivory Coast and Kenya, and the results of interventions on their household income and poverty levels. After reflecting on root causes of poverty, seen through a food systems and living income lens, we then present a new assessment approach to design and tailor policy interventions, and elaborate policy implications for interventions aiming to achieve a living income. Taking a food systems approach allows us to examine poverty drivers to come to conclusions what intervention pathways would be effective in addressing such drivers.

#### We present a new assessment approach to support designing interventions to influence farmer income

This assessment approach shows the type of data to collect and analyse, to provide evidence that can support intervention design, effective in lifting different types of farmers out of poverty. The approach was developed based on evaluations and studies in the tea, cocoa, coffee and other sectors. When used in conjunction with an assessment of the impacts of different interventions on incomes, this provides information on the ability of different interventions to achieve a living income for different groups of farmers. Such evidence enables policymakers and organisations to design more effective and efficient policies and programmes that contribute to achieve living incomes for different types of smallholder commodity farmers.

## Methodology

### Literature review on impacts of commodity program interventions

#### Review of systematic reviews, overview, and meta studies

We reviewed literature to find overview studies on the causes of poverty (guided by the food systems approach) and interventions to improve the income of smallholder tree-crop commodity (cocoa, coffee, palm oil and tea) farmers in lower- and middle-income countries. Literature was collected by asking colleagues about relevant systematic and review studies and searching Google Scholar, as not all relevant studies are published in academic journals. The search criteria included any review or overview study that included evidence on the impact of a specific intervention on crop income, household income or poverty status, in which the counterfactual was addressed, for instance through a comparison group. We searched for studies for each specific crop of interest, as well as for studies that covered multiple crops. Five of such studies were included in the analyses. They addressed technical interventions such as training, standards and certification, input supply, access to finance (credit/loans), contract farming and cash transfers. To better describe the learnings, we also sometimes included information from the articles included in such overview studies. With a deliberate attempt not to present biased information. Overview studies of interventions often combine information from different types of sectors and commodities, so they include a wider range of crops and do not present information solely on interventions covering specific commodity sectors. Therefore, the information from the literature presented in this paper cannot always be connected to a specific sector. An overview of the results of the literature review on the impact of interventions on smallholder commodity farmers is contained in Appendix 1.

#### Very few studies compare actual income levels of smallholder commodity farmers with the world bank poverty line and/or a living income benchmark as most focus on agricultural productivity increase

As the living income concept is relatively new, we did not find any studies that assessed the impact of interventions to close the gap between a living income and actual household incomes. Therefore, we searched for impact evaluations that used the World Bank extreme poverty line to report on (changes in) farmer’s poverty status instead. However, we found that many studies did not focus on measuring a decrease in poverty levels, but rather focused on assessing the impact on productivity or income increase, or the adoption of good agricultural practices. Systematic reviews did not always focus on the impact of interventions on poverty status either.

#### Disaggregation of the impact of interventions by gender or regions was not possible due to scarcity of literature and/or the scarcity of high-quality evaluations

Even though data on the gender of participants in interventions was often collected, gender-disaggregated results on the effects of interventions were not included in the literature reviewed. Also, the effects of interventions on women’s roles and agency in commodity farming were not reported on. Along similar lines, no disaggregated data on interventions in different continents or regions were found, possibly as a result of the low number of high-quality evaluations (see also Bernstein et al., 2019).

#### Most impact evaluations do no not include results on poverty levels of households

Even though we are interested in interventions that deal with the alleviation of smallholder poverty through a food systems perspective, studies that cover those were not found. Therefore, the interventions that were examined are based upon evaluations of interventions that assess the impact on total household incomes. However, many studies focus on commodity incomes rather than total household incomes. Where available, we summarised information from systematic reviews with data on total household incomes, as commodity income increases may not translate in total household income increase. This occurs because of changes in the division of household labour among various income-generating activities. The results presented on the effects on commodity income therefore must be interpreted with caution as they do not incorporate the effects on other sources of income of interventions that may require redistributions of household labour.

### Primary data analysis of household poverty status

#### Data analysis data of farmer’s income levels compared to living income benchmarks

We analysed primary data from three panel datasets generated to evaluate the impact of interventions on the income of smallholder commodity farmers in lower- and middle-income countries:

1. 439 smallholder tea farmers from Kenya: data collected for an impact evaluation study financed and commissioned by KTDA, IDH and Unilever (Waarts et al., 2016). Data are presented for the year 2015.
2. 311 smallholder cocoa farmers from Ghana: data collected for impact evaluation studies financed and commissioned by Solidaridad and UTZ Certified (Waarts et al., 2015). Data are presented for the year 2014.
3. 362 smallholder cocoa farmers from Ivory Coast: data collected for impact evaluation studies financed and commissioned by Solidaridad, UTZ Certified, Cargill, IDH and Nestlé (Ingram et al., 2018). Data are presented for the year 2017.

The farmers in these datasets were seen by programme staff as similar to typical farmers in the cocoa and tea value chain, but our data may have a small bias as half of the sample are programme participants and half of the sample similar farmers to programme participants.

#### Calculating the percentage of farmers against the living income and world bank extreme poverty lines

We used the data from these studies and information from living income and living wage assessments from these countries to calculate the income status with regard to the World Bank poverty line and living income benchmarks to make the results comparable between countries (Anker & Anker, 2015; Smith & Sarpong, 2018; Tyszler et al., 2018). And to show the predicted effects of several interventions (e.g. price increases) on farmers’ poverty status. For the methodology on the calculation of comparable poverty lines and living income benchmarks, please see Appendix 2.

Information on farm characteristics and farmers’ economic status is presented for two groups.

1. Farmers who earn less than the living income benchmark per person per day. In this group, we can distinguish between: i) Farmers who earn less than the World Bank poverty line of USD 1.90 per person per day (2011 PPP) and ii) Farmers who earn between the World Bank poverty line of USD 1.90 per person per day (2011 PPP) and the living income benchmark. This distinction is important to make because many stakeholders work with the World Bank poverty lines, and because this allows for the identification of the poorest and most vulnerable farmers.
2. Farmers who earn the same or more than the living income benchmark per person per day.

Based on this information, we qualitatively assess the potential for different types of commodity farmers to earn a living income, and present an assessment approach that allows, when implemented, to conclude on promising approaches for achieving living incomes for different groups, which are presented and discussed in Sect. 4 on policy implications.

### Literature review on interventions uncommon in commodity programs

After the analysis of poverty drivers and evidence on the impact of interventions on smallholder commodity farmer income (presented in Sect. 3), we conducted a literature search again. The focus of this second literature search was to find evidence on policies and interventions that can directly influence farmer incomes, but which is not commonly discussed in literature on smallholder tree-crop commodity farming, nor addressed by most commodity sector programs. Information on the following policies are included: land governance, social assistance programmes, creating employment opportunities, pricing policies and supply management. Again, we focused on including evidence from overview or review studies, added to by specific articles about the three countries for which we present empirical data: Ghana, Ivory Coast and Kenya.

### Limitations

#### Limitations regarding analyses of environmental drivers, consumer characteristics, and information on interventions not directly influencing household income

Little data was found on the environmental drivers of a food system connected to poverty outcomes and none was found specifically related to the living income concept. We therefore could not conduct in-depth analyses of all environmental drivers impacting poverty and the potential for farmers to earn a living income. Nor did we assess the effects on the environment of increased income. Data paucity in the literature also meant that we did not present analyses on the effects of (changing) consumer characteristics on the potential for farmers to achieve a living income. As the literature scan provided information about the outcomes of interventions on different commodity sectors and countries, the information presented in this paper cannot be connected to one specific sector or geography. Finally, we focused our literature reviews and analyses on cash income. We did reflect on the value of interventions that do not have a (direct) impact on farmers ability to earn a better cash income but did not include an extensive literature review on such interventions.

## Results

### Food system outcome: poverty status of smallholder commodity farmers

#### Most smallholder commodity farmers earn less than the living income benchmark

**Fig. 4:**
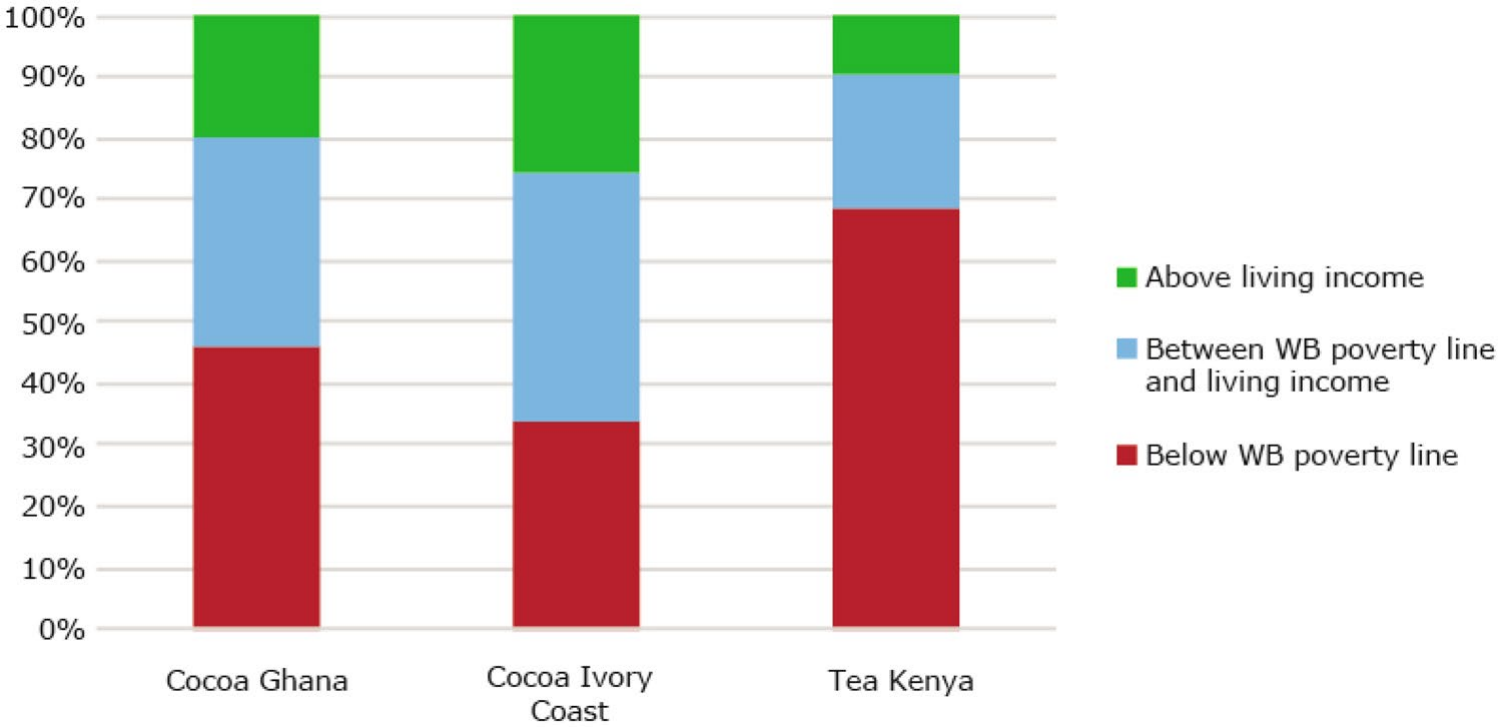
Percentage of smallholder cocoa and tea farmers above and below the USD 1.90 World Bank poverty line and living income benchmarks. Sources: Ghana: (Waarts et al., 2015) (*N* = 311), Côte d’Ivoire: (Ingram et al., 2018) (*N* = 362), Kenya: (Waarts et al., 2016) (*N* = 439)

Shown in Fig. 4, Findings from the three impact evaluation studies revealed that about 82% of cocoa farmers in Ghana and Ivory Coast, and tea farmers in Kenya earned less than a living income at the time of study, and more than half (51%) earn below the World Bank extreme poverty line^2^. In Ghana and Côte d’Ivoire, farmers earn on average about 60% of the living income benchmark. Tea farmers in Kenya earn on average 47% of a living income. This situation of widespread poverty in commodity sectors is confirmed in the literature, irrespective of how poverty is defined (WB poverty line or Living income) (Alvarez & Von Hagen, 2011; Dalberg & Wageningen University, 2018; Oya et al., 2017; Woodhill et al., 2020). The living income benchmarks for the three countries are: USD 1.32 per person per day for tea farmers in Kenya, USD 2.08 per person per day for cocoa farmers in Ghana, and USD 2.52 per person per day for cocoa farmers in Ivory Coast.

### Impact of interventions on household income and poverty levels

#### The effect of interventions on total household income is insufficient for many smallholder commodity farmers to earn a living income even if multi-stakeholder food systems approaches have the best chance of success

**Fig. 5:**
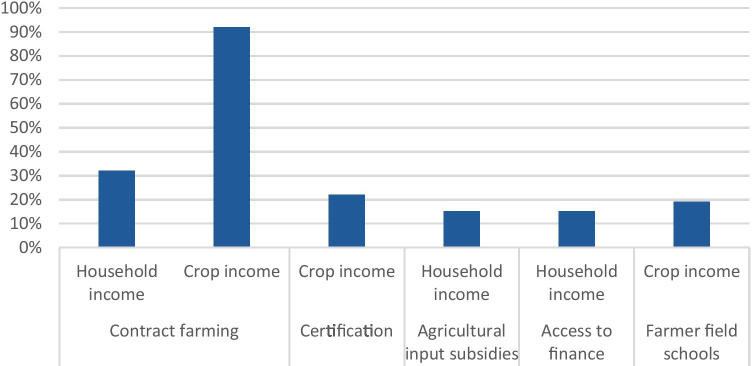
Average percentage increase in crop and/or household income of target group compared to comparison group for different types of interventions (in the information on certification, changes in household income were found to be 13% but were not significant, which is why we did not present this change in the figure). Sources: (Dalberg & Wageningen University, 2018; Hemming et al., 2018; Oya et al., 2017; Ton et al., 2017; Waddington et al., 2014)

Interventions implemented in commodity sectors have between 19 and 90% effect on crop income, and a 15–32% effect on household income (Fig. 5). This information is based on several review studies, information from these different studies can be found in Appendix 1. For interventions on productivity enhancement through training and input services, one study found effects between 10 and 50% (Dalberg & Wageningen University, 2018). A systematic review on cash transfers to individuals or households, reported that six out of nine studies found a significant impact on poverty measures (Bastagli et al., 2016), but also concludes that in many cases the impact is not big enough to have an effect on aggregate poverty levels, and that long term effects are not clear^3^. A recent review study shows that the total household income increases occuring because of diffferent interventions are not enough for poor smallholder commodity farmers to earn a living income, as they may need income increases of 100–200% to do so (Dalberg & Wageningen University, 2018). Another meta evidence review reports that there is ‘insufficient evidence to determine trends’ for the impact of extension and advisory services and agricultural input subsidies on poverty, and that ‘despite evidence, the impact is in doubt’ for improved access to financial products (Bernstein et al., 2019). Multi-stakeholder approaches in which different food system components were addressed have the biggest chance of achieving long-term impact on incomes at scale (Dalberg & Wageningen University, 2018). However, the impact of many interventions is often not big enough for the poorest farmers to earn a living income.

#### Positive impacts on income in studies to be interpreted with caution because of study and target group biases

Various biases in the literature (‘survival bias’, ‘publication bias’, ‘selection bias’ and the fact that not many peer reviewed studies have been conducted) means that the evidence in the literature reviewed most likely overestimates the impact of interventions on income. The reasons for this are that: i) Evidence of projects and programmes that stopped in their early years is generally not collected and/or published, ii) Farmers included in interventions are not necessarily representative of all farmers in the sector, e.g. the poorest farmers may not participate, iii) Farmers who have dropped out of an intervention are often not included in research after they left the project and iv) If study outcomes are not significant, they are less likely to be published (Ton et al., 2017). Also, the number of academic peer reviewed studies containing evidence on the topic are low.

### Root causes of poverty levels

#### Socio-economic drivers and the enabling environment

##### Farm sizes are often too small to earn a living income, and are likely to decrease due to inheritance structures

**Fig. 6:**
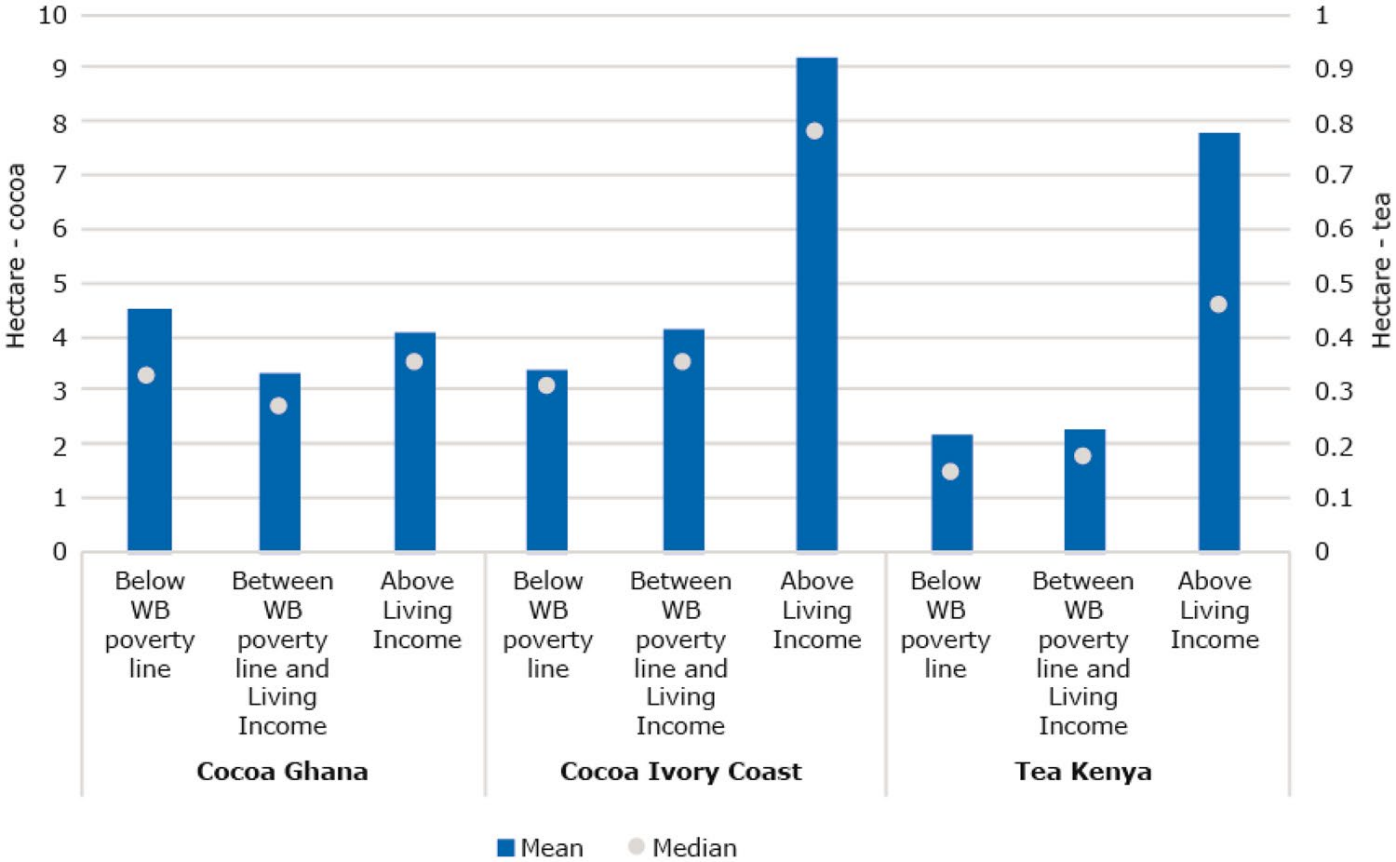
Mean farm sizes in hectare for different groups of cocoa farmers in Ghana and Ivory Coast and tea farmers in Kenya. Sources: Ghana: (Waarts et al., 2015) (*N* = 311), Côte d’Ivoire: (Ingram et al., 2018) (*N* = 362), Kenya: (Waarts et al., 2016) (*N* = 439)

Small farm sizes can be a key driver of poverty. In Ivory Coast and Kenya, the poorest farmers in the datasets have the smallest farm sizes, 3–4 hectares and about 0.2 hectares on average respectively (Fig. 6); this is also confirmed by another study on the cocoa sector in Ghana and Ivory Coast (van Vliet et al., n.d.). Tea farmers in Kenya have much smaller farm sizes than cocoa farmers in West Africa, but they earn relatively more per hectare than cocoa farmers because they harvest every week to every two weeks instead of twice a year. Land fragmentation is also confirmed by the literature as a driver of poverty (Giller et al., n.d.). studies, minimum farm sizes for ‘economically viable’ farms are calculated, but we find that such farm sizes are not necessarily minimum farm sizes for earning a living income. One study presents that the minimum economic tea farm unit for smallholder farmers in Kenya is 0.1 ha (0.25 acres) (Kavoi et al., 2002). But looking at the datasets, households with such small farm sizes are extremely likely not to earn a living income. In the dataset on cocoa farmers in Ghana, we do not find that poorer farmers generally have smaller farms, but find other important factors influencing poverty levels which are presented below. Small farm size does not have to be an impediment for earning sufficient incomes in all sectors but remains an important factor to consider.

##### Farm sizes would need to at least double to enable the cocoa and tea farmers, who currently earn below the living income, to earn a living income

**Fig. 7:**
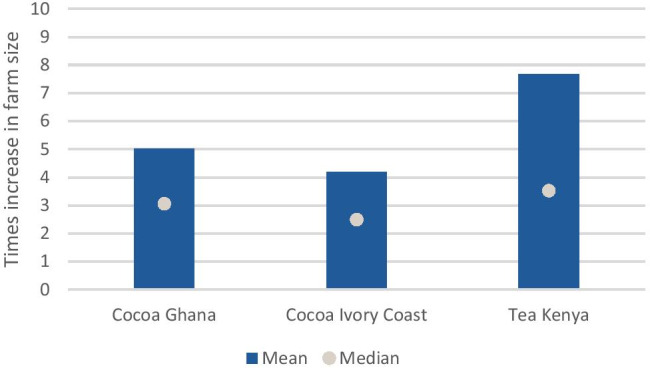
Increase in current farm sizes needed to close the living income gap for farmers who currently earn less than the living income benchmark. Sources: Ghana: (Waarts et al., 2015) (*N* = 311), Côte d’Ivoire: (Ingram et al., 2018) (*N* = 362), Kenya: (Waarts et al., 2016) (*N* = 439)

For farmers to achieve a living income – when all other variables remain constant – farm sizes would need to increase significantly, ranging from an increase of four in Ivory Coast to almost eight times in Kenya (see Fig. 7). Interestingly, farm size in Ghana would need to increase more than in Ivory Coast while we saw earlier that farm size is less of a barrier to earning a living income in Ghana than in Ivory Coast. Given that some of the main factors for land fragmentation globally are population growth and inheritance (Demetriou, 2013), and the population of many lower and middle income countries is still growing rapidly^4^, it is unlikely that such increases in farm size can be achieved easily.

##### Possibilities for income diversification are limited; farmers are very dependent on cocoa or tea as their main source of income

**Fig. 8:**
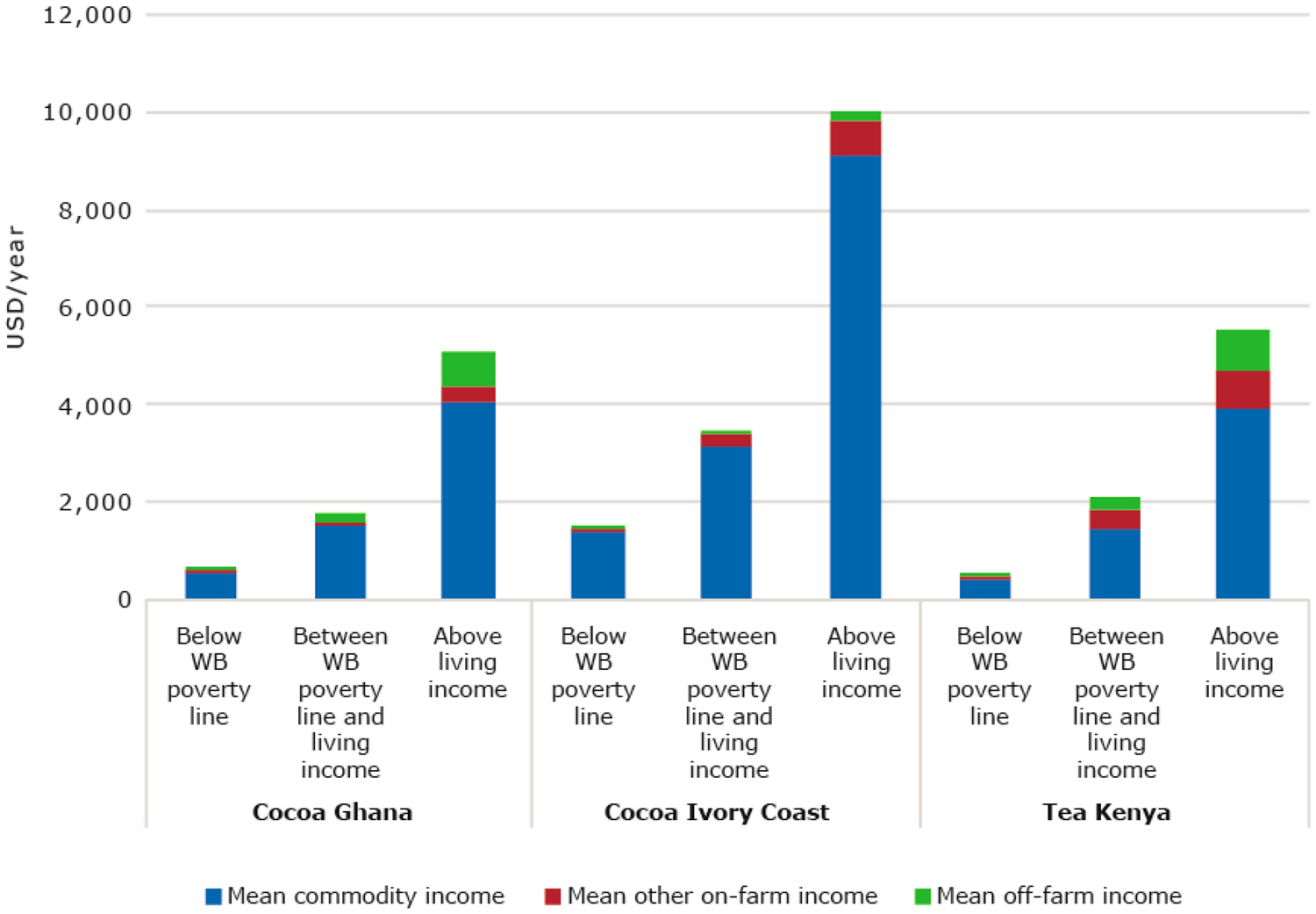
Income earned per household member per year (USD Purchasing Power Parity) (For comparison, the monthly living income line per family was converted to a daily living income per household member). Sources: Ghana: (Waarts et al., 2015) (*N* = 311), Côte d’Ivoire: (Ingram et al., 2018) (*N* = 362), Kenya: (Waarts et al., 2016) (*N* = 439)

Farmers are very dependent on cocoa and tea as their main source of income as they earn most of their income from the commodity crop (Fig. 8). For example, cocoa farmers in Ghana who earn more than a living income (20% of all farmers), earn about USD 5000 per year, of which about USD 4000 comes from cocoa (79%). Cocoa farmers in Ghana who earn less than the World Bank poverty line (46% of all farmers) earn on average about USD 600 per year of which about USD 500 from cocoa (84%). Differences in dependency on the commodity crop are small between the groups. Income diversification is a challenge for these farmers as opportunities are not available or are not rewarding enough compared to commodity production given the current circumstances, human resource assets, and their ability to access investment credit that have affordable (low) interest rates. Access to credit, market linkages and the availability of pro-poor options for conservation, is what drives farmers’ incentives and decisions (Shiferaw et al., 2009). At the same time, diversification is often considered as having promising results, also to increase resilience, especially for the poorest farmers (Asfaw et al., 2019).

##### Commodity market prices are generally volatile and higher prices have not lifted large numbers of farmers out of poverty

Global commodity market prices are generally volatile and cannot easily be set by producing countries, and even then hve not resulted in persistently higher incomes (Bymolt et al., 2018; Squicciarini & Swinnen, 2016). Increasing farm-level buying prices is one of the mechanisms commonly proposed to increase farmer incomes, especially for cocoa, as prices have effectively declined over the past three decades for conventional cocoa (Tröster et al., 2019; Squicciarini & Swinnen, 2016). Tea prices have decreased since 2013, ‘although they remained much higher than the historical average over the previous two decades, both in nominal and real terms’ (Chang, 2007). Higher prices, for example paid by some specialty and certified cocoa buyers can contribute to increase farmer incomes but have not yet lifted large numbers of farmers out of poverty (Ingram, 2014; Purcell, 2018; Tony’s Chocolonely, 2020).

##### Even significant price increases would not achieve living incomes for the poorest tree-crop commodity farmers; relatively richer farmers benefit more because they produce larger volumes

**Fig. 9:**
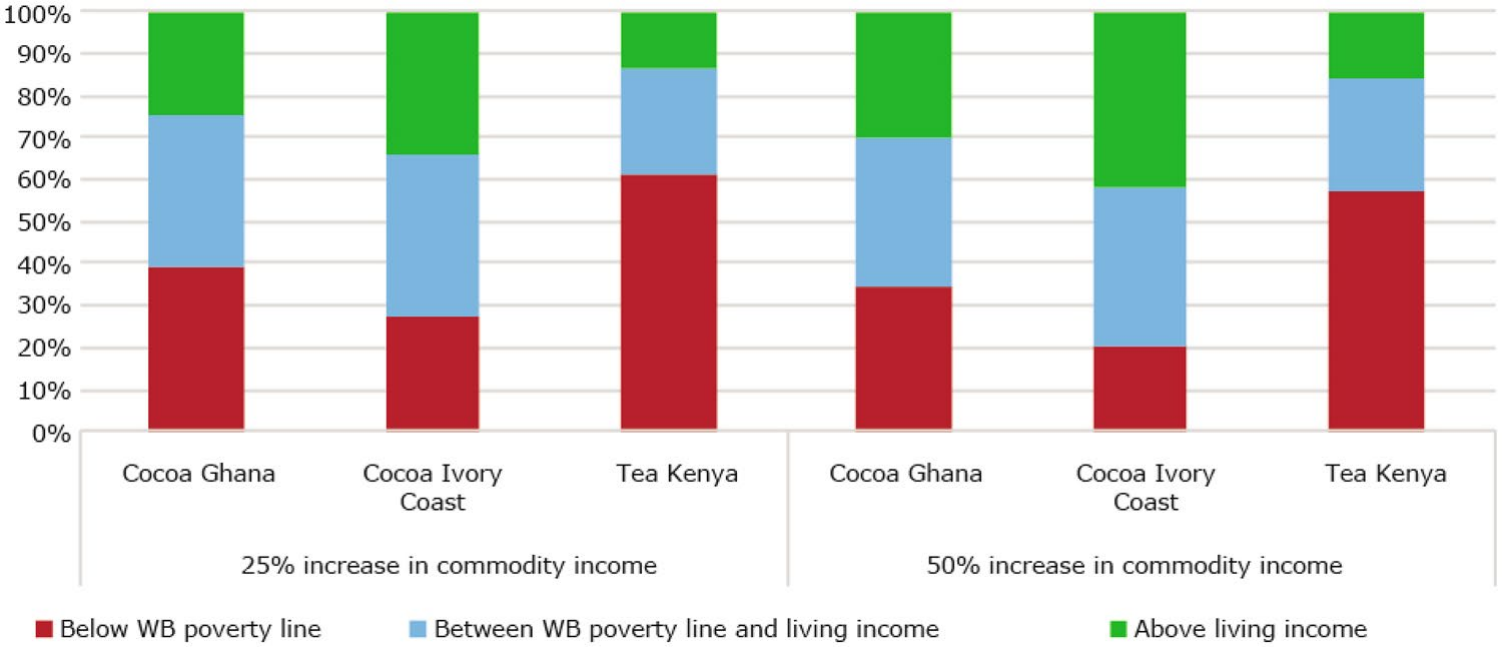
Scenarios for the impact of price increases on percentage of smallholder cocoa and tea farmers above and below the USD 1.90 World Bank poverty line and living income benchmarks (a 50% price increase is assumed to lead to 50% income increase). Sources: Ghana: (Waarts et al., 2015) (*N* = 311), Côte d’Ivoire: (Ingram et al., 2018) (*N* = 362), Kenya: (Waarts et al., 2016) (*N* = 439)

An example where governments influence cocoa farm-gate prices is the Living Income Differential established by the governments of Ivory Coast and Ghana to be paid for cocoa from the 2020/2021 season onwards (Angel et al., 2019; Vidzraku, 2018). It amounts to USD 400/Mt cocoa on top of the FOB-price, an increase of 16% based on a market price of USD 2501/Mt (ICCO, 2020) if it is fully transferred to the farmers. A 16% price increase would have resulted in about USD 13 additional income per household member per year for the poorest Ghanaian cocoa farmers in our study, who earn less than the living income benchmark as well as less than the World Bank poverty line (see Appendix 3). The minimum Sustainability Differential of USD 70/Mt for cocoa as of July 2022, paid as part of the Rainforest Alliance, 2020 certification programme (Rainforest Alliance, 2020), would have increased incomes by about USD 2.5 per household member per year for the same group of farmers (see Appendix 3), and it would benefit only Rainforest Alliance certified farmers. Even though every additional dollar earned is important for farmers, the benefits of price increases are limited in terms of poverty reduction at scale, as the poorest farmers benefit the least because they produce the lowest volumes. This is also confirmed by a study of cocoa farmers in Ghana and Ivory Coast (van Vliet et al., n.d.): with a 50% income increase, 30% of cocoa farmers in Ghana earn above a living income, compared to 20% without the price increase (Fig. 9).

##### Price increases may induce oversupply and indirect negative effects on the environment and may lead to companies changing their sourcing strategies. Price increases should therefore be combined with other measures if implemented at scale

Price increases generally influence farmers to invest in commodity production, leading to production and supply increase, putting a downward pressure on prices again in the long run if demand for commodities does not increase at the same pace (Waarts et al., 2019). If countries increase prices, demand for produce from the country could also decrease if buyers decide to buy their produce elsewhere because of lower prices. Incentives that increase cocoa production can enhance deforestation when commodities have elastic demand in the short term (i.e. the price does not decrease when supply increases) such as is the case for many commodities (Abbott et al., 2005; Tothmihaly, 2017). The legal and financial, for example anticompetition and trust law implications of fixing markets prices also need consideration. Additional measures are thus critical if prices increases are to contribute to achieve living incomes and alleviate persistent poverty, ensure no negative effects, externalities, both in country but also tele-coupled impacts globally materialise. This is discussed further in Sect. 4.1.6.

#### Food supply system, business services and environmental drivers

##### Various factors lead to low adoption rates of good agricultural practices and thus lower incomes

**Fig. 10:**
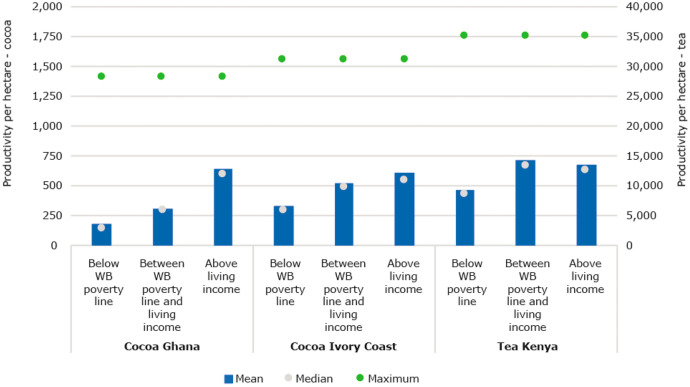
Productivity per hectare per farmer group and the maximum yield level in the research area confirmed by experts. Sources: Ghana: (Waarts et al., 2015) (*N* = 311), Côte d’Ivoire: (Ingram et al., 2018) (*N* = 362), Kenya: (Waarts et al., 2016) (*N* = 439)

Farmers often decide not to adopt new technologies and change their farm management practices and are seen to dis-adopt after initially adopting new practices or technologies. Reasons for low adoption are: i) Failing markets for instance for inputs; ii) Interventions which are not tailored to aspirations, needs and opportunities; iii) Inability to invest time and money, because they do not have the funds and/or a lack of access to affordable credit for instance because of low market prices, and; iv) Investment benefits are not guaranteed leading to financial risk (Bulte et al., 2014; Conley & Udry, 2010; Greiner et al., 2009; Prokopy et al., 2008; Waarts et al., 2019). Low adoption levels or farming in unfavourable circumstances regarding agro-ecological conditions lead to low yield levels and thus to incomes that are lower than what would be feasible. This is also experienced by cocoa farmers in Ghana and Ivory Coast. Farmers under-invest in implementing farm management practices, which leads to a ‘low input-low output’ system (Fig. 10). Low yields are attributed to low input use, inadequate weeding and farm maintenance, insufficient pest and disease control, poor shade management, low rates of fertiliser use, and the old age of some cocoa farms (Bymolt et al., 2018; Wessel & Quist-Wessel, 2015).

##### Farmer’s large yield gaps can be decreased by addressing underlying reasons for low adoption rates of good agricultural practices

Productivity levels for cocoa and tea farmers are low compared to what is possible in the study region (Fig. 10). But these productivity levels are especially low for the poorest farmers. A yield gap cannot be easily and quickly closed; there is a good reason why it exists: adoption of new practices can only lead to improved agricultural productivity when conditions and circumstances are right. Farmers are limited by the environmental drivers in the food system (e.g. low soil quality and unpredictable and heavy rains). Mitigating the effects on their livelihoods of these environmental drivers requires the adoption of good agricultural practices, which in turn requires addressing the key reasons for low adoption rates as listed above.

### New assessment approach to design and tailor policy interventions

#### Introduction to the new approach

##### A new assessment approach to assess the potential of farmers to earn a living income, and design short- and medium-term interventions that address drivers of poverty

**Fig. 11:**
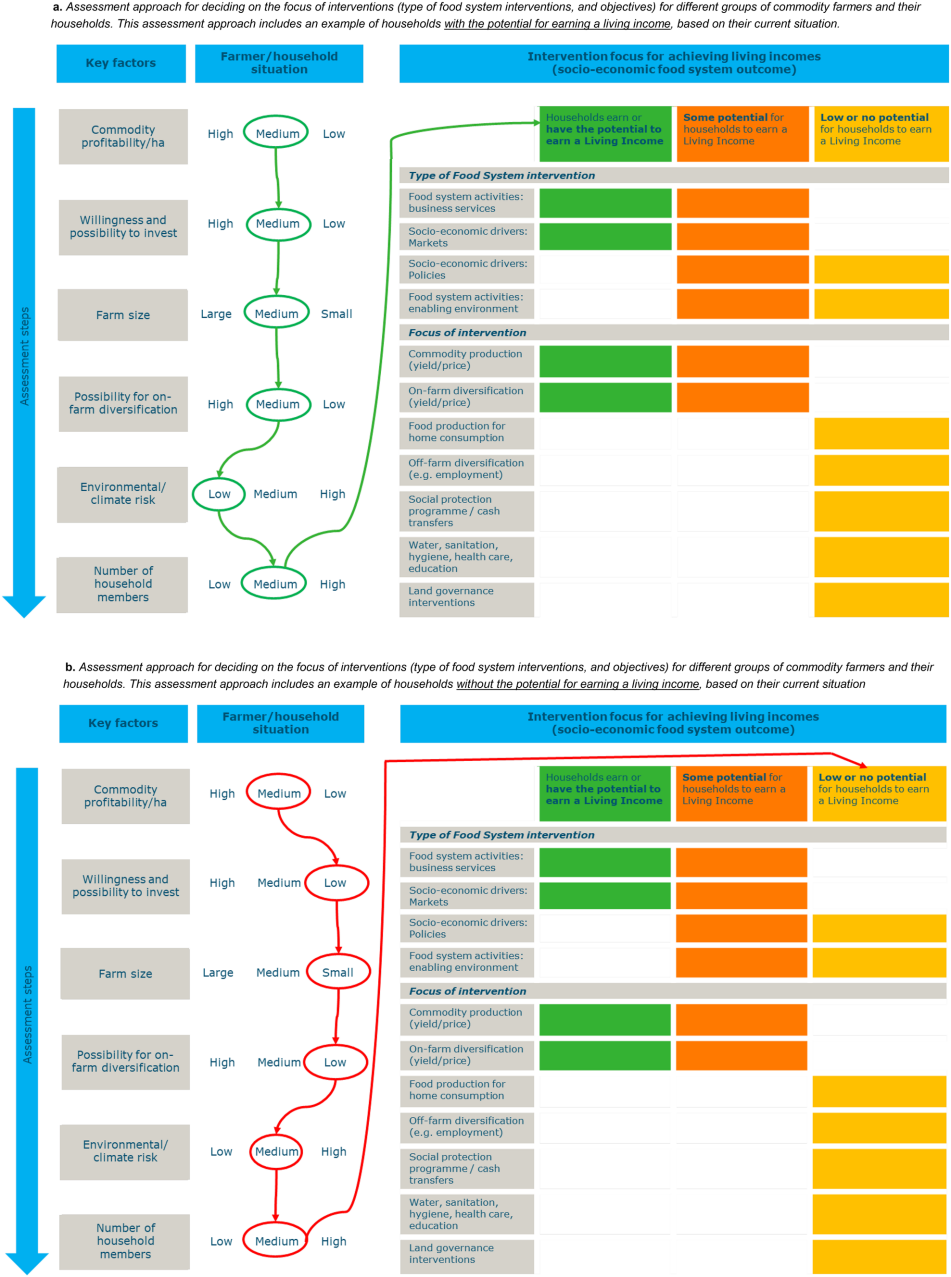
**a** Assessment approach for deciding on the focus of interventions (type of food system interventions, and objectives) for different groups of commodity farmers and their households. This assessment approach includes an example of households with the potential for earning a living income, based on their current situation. **b** Assessment approach for deciding on the focus of interventions (type of food system interventions, and objectives) for different groups of commodity farmers and their households. This assessment approach includes an example of households without the potential for earning a living income, based on their current situation

A first step in intervention design is to assess which smallholder commodity farmers in a certain area and value chain have the potential to earn a living income based on their current conditions, and which do not, and why this is the case. This can be a complex task because multiple factors influence the potential for farmers to earn a living income. To support such a design process, we present an assessment approach which can be used to design short- and medium-term interventions. This approach was developed based on our empirical research work in commodity sectors^45^ as well as the literature, and inspired by the ‘pathways to prosperity’ report (Shakhovskoy et al., 2019). It presents the key determinants influencing the ability of farmers to earn a living income and it can be used in different smallholder commodity contexts. It does not provide detailed thresholds for each factor per sector and/or contexts, but it is a tool for policymakers and private sector to understand about the farmers they work with and/or buy from. The assessment approach is elaborated below and shown in Fig. 11a, b.

##### Six factors need to be assessed, to decide on intervention design focus

From our empirical studies, we found six key factors that need to be assessed to support intervention design focus:

1. Commodity profitability per hectare, which includes production volumes, yield per hectare, prices received, and cost of production
2. Farmers’ willingness and possibility to invest, including whether there is affordable credit available
3. Farm size
4. Possibility for on-farm diversification, including business activities performed at the homestead/farm.
5. Environmental or climate risk and
6. The number of household members^5^.

Information on these six factors should be analysed in combination with each other for a farmer or group of farmers, to come to conclusions what intervention focus would be most effective. For instance, a farmer may have a very high profitability per hectare without the chance to improve performance further, but because of small farm size she cannot earn a living income, except when a household member would be employed elsewhere or when a business is established. Based on information on possibility to invest, market aspects and employment prospects, a decision can be made together with the farmer what intervention(s) would work best. Such analyses and discussions can also be conducted for groups of farmers.

##### Because smallholder commodity farmers are generally quite dependent on income from the commodity, and productivity per hectare and profitability is often low, cocoa profitability is the starting point of the analyses

Commodity production is generally a first point of entry for many interventions as they focus for a large part on improving productivity because of large yield gaps, and because the majority is highly depending on income from the commodity. But without the possibility to invest in improving profitability, commodity income cannot increase. Therefore, a second factor to be assessed is the willingness and possibility to make financial investments in farming or other activities. If farmers do not have the means to invest money (through cash, savings, or credit) or are not willing to do so because they need to/choose to spend their money elsewhere (e.g. funerals, education), it will be hard to increase incomes greatly. Such ‘willingness’ also includes various contextual and personal factors influencing decision making (see Sect. 1.3).

##### After assessing farm size and whether and how the farm size hampers income increase, the possibility for on-farm diversification for the generation of cash income is assessed

When enough land is available and the farmer is willing and able to invest, but there is no input supply or market demand for alternative crops or supply chains do not function, it will be a challenge to diversify (this also includes relevant contextual factors enabling diversification, see Sect. 4.2). Also, future expectations regarding agro-ecological and climatic conditions are important to be considered to decide whether and how to invest time and money.

##### When income from farming does not enable farmers to achieve a living income, other ways should be found to strengthen their income and resilience in the short term

There is a possibility that farmers cannot increase their income substantially enough for achieving a living income. Because they do not have enough land, money or time to invest, because markets are failing, or because environmental circumstances hamper production. We focused our analyses on cash income as the living income calculations are about the cash income needed for a decent standard of living, but realise that there are farmers who do not have the potential to earn a living income. This consideration needs to be taken into account in the living income assessment approach as in that case such farmers need different types of support for livelihood improvements. Examples are employment facilitation for direct income effects, helping farmers and their households to become food secure, ensuring children are educated, that health care is accessible as well as safe water and proper sanitation, and by helping people to apply proper hygiene practices. While for all farmers this is important, such support is especially important for farmers without the potential to earn a living income.

##### When large numbers of households do not have the potential to earn a living income, long term policies around land governance, employment creation and/or cash transfers/social welfare payments are needed

Finally, the assessment approach results may conclude that the economic situation of the large numbers of farmers and their households cannot be improved within their current circumstances (available land, willingness and possibility to invest, possibility for yield improvement and diversification). In that case national, regional or landscape level policies are required that address the structural factors underlying poverty levels such as land fragmentation, employment opportunities and price volatility.

##### Five possible intervention categories identified as a result of the living income assessment approach

Based on empirical evidence and our experience, the living income assessment approach results in five possible intervention categories built on the potential for farmers to earn a living income and types of food system interventions to do so (see also Fig. 11a, b):

1. Farmers who earn a living income or have the potential to do so: Interventions to focus on improving income from commodity production. This may include yield or price improvements, cost reductions or a combination.
2. Farmers who earn a living income or have the potential to do so. Interventions to focus on improving income from on-farm diversification, possibly in combination with improving commodity income. This may include yield or price improvements, cost reductions or a combination.
3. Farmers who have some potential for earning a living income: Interventions to focus on improving income from commodity production. This may include yield or price improvements, cost reductions or a combination.
4. Farmers who have some potential for earning a living income: Interventions to focus on improving income from on-farm diversification, possibly in combination with improving commodity income. This may include yield or price improvements, cost reductions or a combination.
5. Farmers with a low or no potential to earn a living income: interventions to focus on the following support, depending on needs and possibilities:
  - Off-farm diversification, such as employment
  - Food production for home consumption, to improve food and nutrition security
  - Water, sanitation, hygiene, healthcare and education
  - Social protection programmes.
  - Land governance interventions when many farmers have too small farm sizes hampering them to earn a living income.

#### Assessment approach results based on empirical data presented earlier

##### A large proportion of households in our data are estimated to have a low potential to earn a living income. They need different support than farmers who have the potential for earning a living income

Interventions aimed at commodity profitability improvement are suitable for farmers who have the potential to earn a living income based on their current conditions. For instance, because their profitability can be improved, they have medium to high willingness and possibility to invest, they have enough land, and environmental/climate risk is low. In a situation where such farmers also have the possibility to earn additional income though diversification, such farmers can also be supported in on-farm diversification, depending on farmers’ aspirations as well as the expected benefits of both options. For more information on how to implement such support activities with these farmers, please see Sect. 4.2. In Ghana, Ivory Coast and Kenya, 20%, 26% and 10% of the farmers in our data earn a living income respectively. We cannot calculate the exact proportion of farmers who have the potential to earn a living income, but based upon our data, the literature and experience, we conclude that a large proportion of households does not have the potential to earn a living income without policies that address the structural factors underlying poverty such as employment, land fragmentation, price volatility and social protection programs.

#### Connecting living income assessment approach results to policy implications for intervention design

##### The food systems approach is used as a lens to look at interventions that may contribute to achieving living incomes, focusing on interventions supporting the most vulnerable farmers

In designing interventions aiming to lift smallholder commodity farmers out of poverty, it is important to take a food systems approach for the interventions to be effective and not a commodity production approach. Therefore, we use the food systems components to discuss evidence on different interventions and how they could contribute to achieve a living income based on the literature in Sect. 4. In the living income assessment approach, we start the analyses with commodity profitability as commodity farmers largely depend on commodities for their income, and governments, companies and NGOs also often have a commodity focus. The five intervention categories defined as a result of our living income assessment approach are integrated in the discussion on policy implications in Sect. 4. This integration is also depicted in Fig. 11a, b^6^. We start the discussion on the interventions with interventions addressing socio-economic drivers and the enabling environment (Fig. 2). This because a large proportion of farmers in our datasets have a low or no potential to earn a living income based on their current conditions and are therefore in need of such interventions. We cannot recommend which intervention would be best suited, and which interventions should be prioritised, as the contexts in which smallholder tree-crop commodity farmers operate differ widely between countries and within countries. Therefore, based on the results of the living income assessment approach, organisations together with farmers, households and communities should decide on what interventions are most suited.

## Policy and practical implications of interventions to achieve a living income

### Interventions addressing socio-economic drivers and the enabling environment

#### ‘One health’ interventions

‘One health’ type interventions in for example water, sanitation, hygiene, health care, food and nutritional security (Arsyad et al., 2019; Walton et al., 2020) combined with education can be important in alleviating poverty. This is especially in cases where farmers do not have the potential in the short and medium term to earn a living income from their main cash crop. Interventions for these farmers could focus supporting farmers and their communities to improve their living conditions and the environment. As we focus in this paper on the living income concept, and therefore on interventions with a direct sphere of influence on increasing incomes, further details of such interventions are out of scope of this paper.

#### Addressing land fragmentation: policies aimed at formalisation and privatisation of land tenure

##### Policies on land governance and job creation enable people to improve their situation and/or move out of self-employed farming. If some farmers ‘move out’, this may enable remaining farmers to earn a living income

Land fragmentation is a challenge in Sub-Saharan Africa, with ‘a vast majority of farms far less than 1 hectare’ and there are expectations that farm sizes will further decrease due to the increasing population in connection with inheritance structures (Giller et al., n.d.). To prevent increases in poverty due to land fragmentation, policies are needed on land governance and employment creation that support people to improve their situation with farming or move out of self-employed farming into other activities. People finding employment elsewhere may give remaining farmers the opportunity to work on a profitable farm that enables them to earn a living income. Such a process should be properly implemented to avoid human rights violations. To achieve shifts of labour to other sectors it is important that the enabling environment is correctly set up for this. Policies to be analysed for improvement are: tenure and land use planning (e.g. on farm ownership and minimum farm size) and inheritance structures and policies.

##### Different land tenure systems determine how access to and control over land is governed and are therefore of key importance for land use planning

Land tenure systems in sub-Saharan Africa can generally be divided into three main categories: private land, public (state-owned) land and community land (customary tenure)^7^. Both private land and public land are governed by statutory law, as opposed to customary law where land is often communally held and transferred by a ‘traditional’ law of succession rather than formal transfer of title (Atwood, 1990)^8^. Different tenure systems often co-exist and in some cases overlap. Many countries, including Kenya, Ghana and Côte d’Ivoire, have a plural tenure system including forms of private, public, and customary land tenure (Putzel et al., 2015)^9^.

##### In a response to drastic political and socio-economic changes that have impacted land use and increasing pressure on land in Sub-Saharan Africa, many countries have initiated land reforms in the past twenty years

Policy discussions since the 1990s have reflected conflicting interests and different visions of legal reform around land. These can broadly be divided into three categories: i) the agenda centred on formalisation, registration and promotion of private property rights; ii) efforts to institutionalise smallholder user rights and iii) reinforcing community land rights (Boone, 2019). In line with the first agenda, also advocated by the World Bank, many African countries have adopted land law reforms that aim at individual registration and land titling, which brings farmers who were previously operating under customary law under a private/modern tenure system. Examples are Kenya’s land reform in 2012 and Côte d’Ivoire in 1998 and 2015. The objective of privatising farmland has been to integrate smallholders into the market economy and to create opportunities for investment, modernisation and upscaling.

##### The formalisation of land rights may negatively affect vulnerable groups

Land registration is often advocated as a pro-poor empowerment strategy, and ‘some see registration and titling as a way to protect smallholders’ rights of access to land’ (Boone, 2019). However, the formalisation of land rights can generate a number of tensions and trade-offs. For example, it can expose poor and vulnerable groups to adverse market effects. Markets potentially expose poor farmers and vulnerable groups to high risks of dispossession, as the process of individualisation and formalisation of land titling does not recognise all existing forms of land use and land ownership (Chang, 2007). Formalisation often materialises as top-down restructuring and involves risks including elite capture (Putzel et al., 2015). The process of formalisation may actually solidify practices that negatively affect vulnerable groups, including women, youth, ethnic minorities or land-users that do not own land (AFD Land Tenure & Development Technical Committee, 2015; Notess et al., 2020). In addition, changes that erode communal structures enhance the economic autonomy of individuals vis-à-vis extended families, community leaders, or the community at large, which creates individual opportunities but might also have larger socially-disruptive effects (Boone, 2019).

#### Addressing land governance: fragmentation, land rights protection, and communal approaches to agricultural production

##### As a response to the market-led dispossession of smallholder African farmers, an option is to secure smallholder farmers land rights

Advocates of this approach argue that user-rights securitisation would protect the poor from arbitrary dispossession by government, powerful elites, and other so-called ‘land-grabbers’ (Stein & Cunningham, 2017). Programmes have also focused on reducing disruptive land conflicts and strengthening the position of women. Registration that aims at securing smallholder user-rights often involve local-level land administration and governance institutions empowering rural communities and their members to govern their own assets locally. A question is how these regimes are sustained over time in the face of changes such as growth of extended families, on-going socio-economic differentiation or adverse shifts in national and international regulatory contexts for smallholder agriculture (Boone, 2019). The importance of tenure security for smallholder farmers and other vulnerable groups of land-users is now widely acknowledged by scholars, policymakers and practitioners.

##### …or strengthening of communal land rights

Rather than individual titling, this would mean the legal recognition of local communities’ collective right to own customary tenured lands, with guaranteed full legal protection as private land-owners. Potential tension or trade-offs of such schemes are the extent to which they compromise (national) democratic institutions and solidify the power of local elites. In addition, formalising community ownership has the potential effect of ‘hardening’ group identities and artificially creating group boundaries, by formalising who belongs or does not belong to a particular group or community (Boone, 2019; Putzel et al., 2015). In a recent example, six communities in Lofa County, Liberia, have been certified as land-owning communities by the Liberia Land Authority in 2020 (FPA, 2020). This means the communities now govern and manage their land collectively, according to their own by-laws administered by a representative local body. It will be interesting to learn whether the expected benefits of this process, such as forest protection and the improvement of livelihoods (IDH, n.d.), indeed materialise on a landscape scale.

##### Alternative land use governance mechanisms such as ‘block farming’ could lead to better incomes, though there is a lack of evidence on their impact on commodity farmer incomes

A mechanism that may increase farmer incomes through efficient labour division and cost-effective service delivery is 'block farming’, which has been implemented in commodity sectors in the past two decades. Collaboration between farmers and with the first buyer can create economies of scale in both production and service delivery. Various forms of block farming exist. In a first model, the processor owns or has full control of the land (e.g. titles), farm management is done collectively and farmers are paid for what they produce (Ugwu, 2020) or are paid wages while also sharing in the enterprise’s risks and rewards as part-owners (PEF, 2016). A second model is one where a group of farmers owns the land title and works together to optimise costs (Kimbugwe, 2020). A third model is one in which individual farmers own land titles and manage their farms individually while collaborating with other farmers (Pantoja et al., 2019). Even though we find positive information on such mechanisms’ impact on income, we did not find evidence based on academic research standards that (all participating) farmer incomes improved and whether they improved enough for most farmers to be lifted out of poverty (Department of Agrarian Reform, 2019; IFPRI Ghana, n.d.; Matenga, 2017; Nicavera, 2018; Pantoja et al., 2019).

##### Potential unintended negative effects of ‘block farming’

Block farming is often described as a mutually beneficial relationship between processing companies and smallholder farmers – but there are some trade-offs, such as a possible refiguring of social relations (Matenga, 2017) and gender imbalance. There can also be longer-term risks or tensions associated with the loss of control/ownership over land. A well-implemented block farming model programme has the potential to provide a sustainable supply of raw materials to the processor while at the same time improve income and livelihoods for smallholders. For block farming models to work, land titling is seen as crucial, but defining the bounds of tenure and individual/communal ownership can be a challenge (Kimbugwe, 2020).

##### There is no ‘one size fits all’ solution: each approach to land governance has potential tensions and trade-offs

What is an appropriate sustainable and rights-based solution to land fragmentation is a highly contextual question. This requires attention to the existing land use and land ownership practices, power asymmetries and inequalities based on gender, lineage, age, group membership that are already embedded in existing land ownership/inheritance structures and (informal) land use practices. This also requires attention to the social undesirability of selling of or leasing out inherited land. Also, the expected impacts of such solutions should be assessed. It is equally important to recognise the dynamic aspirations of all the people in a certain area including the poorest, the diversity among them, and different options for livelihood diversification, as presented in Fig. 8. Also, climate change forecasts and demographic trends such as urbanisation, and forest and biodiversity protection targets should be integrated in creating new land use plans.

#### Alternative employment opportunities

##### Alternative employment opportunities need to provide better options than self-employed farming for people who decide to earn an income other than from commodity crops. But earning a living wage is generally not guaranteed in many sectors

If alternative employment opportunities are not considered better compared to self-employed farming, people might not be willing to switch, even when switching to other activities may allow them to obtain additional income from renting out their land. There is no guarantee that switching to other activities will lead to earning a living income. Living wage benchmarks are generally much higher than minimum wages and prevailing wages (Global living wage coalition, 2020). For instance, the living wage benchmark for the banana sector in Ghana finds that, on average, workers are paid 74% of the living wage estimate (Smith & Sarpong, 2018), whereas households engaged in self-employed farming in the cocoa sector on average only earn 48% of the living income benchmark (Smith et al., 2017). This indicates that in Ghana cocoa farmers might be better off financially when they switch to wage labour in the banana sector. Also, though, it indicates that such a switch does not guarantee them to earn a decent income.

##### Creating new and decent employment opportunities has proven to be difficult

Regardless of the relatively high economic growth that has been experienced by African countries, growth in decent employment was very low (Yaïche, 2019). This is partly a result of the unsuccessfulness of structural transformation, characterised by the limited contribution of the manufacturing sector to economic growth (Yaïche, 2019). Many projects with the aim of creating new job opportunities have focused on small firms. Although small firms may grow faster than large firms after surviving the first couple of years, when survival rates are taken into account (about half of the firms no longer exists after 3 years), expected job growth for large and small firms does not significantly differ. Additionally, wages are much higher in larger firms (Page & Söderbom, 2015). An example from the tea sector in Malawi shows that thanks to a collaboration since 2015 between plantations, unions, tea buyers and the government, the gap to a living wage was decreased. But living wages have not been achieved yet because of inflation eroding the value of wage increases and the exchange rate between the US Dollar and the Malawi Kwacha amongst others (Chiwaula et al., 2020).

##### Improving education and infrastructure could benefit the creation of off-farm employment opportunities and agricultural production

To stimulate labour transfers out of self-employed farming, policy makers may wish to consider relevant enablers and barriers. Investments in employment creation should not focus solely on supporting small firms, but should i) focus on removing the constraints for firms to grow, ii) identify firms with a high potential to survive, and iii) support firms not only in terms of finance but also capacities (Page & Söderbom, 2015). Firms of all sizes report that infrastructure deficiencies (electricity and transportation) are the most important barriers to growth (Page & Söderbom, 2015), which would also be key leverage points for the adoption of better agricultural practices and increasing agricultural productivity (Page & Shimeles, 2015). Access to some public and private assets (including education) are generally advocated as enablers to increase non-farm activities of rural households (mainly through self-employment), whereas being credit constrained or experiencing poor infrastructure or poor locations may hinder non-farm self-employment (Dedehouanou et al., 2018). A focus on private sector development in agro-processing, manufacturing and tradeable services, with a focus on the export sector, is needed to create more and better jobs (Page & Shimeles, 2015).

#### Social assistance programmes and cash transfers

##### Cash transfers and basic income interventions are tested and proposed to decrease poverty

Currently, social protection systems are weak in Sub-Saharan Africa and, if they exist, they ‘tend to benefit mostly formal workers’ (Molina & Ortiz-Juarez, 2020) and thus generally do not benefit smallholder farmers and their households. Social protection systems, and specifically social assistance programmes including cash transfers, could improve the income of those poor smallholder commodity farmers in Sub-Saharan Africa who do not have the potential to improve their income. Social assistance systems are currently specifically called for because the Covid-19 outbreak creates ‘devastating costs for the livelihoods of less advantaged people’ (Molina & Ortiz-Juarez, 2020). Cash transfers can be unconditional or can contain behavioural conditions for receiving the payment but often are implemented for a short duration. More recently Universal Basic Income schemes^10^ were started or are prepared, and a Universal Ultra Basic income has been proposed (Banerjee & Duflo, 2019; Molina & Ortiz-Juarez, 2020). The payment of Temporary Basic Income (TBI) has been proposed as a response to the effects of Covid-19. There can be differences between the interventions in: who is targeted (all or only the poor? Only certain age groups?), who in a household receives the income (each individual or one person for the entire household?), and the height of the payments.

##### There are different ways in calculating the height of the payments

The ILO indicates that the benefit level of the Universal Basic Income should at least ensure a basic standard of living^11^ for those who do not have another source of income: ‘If benefit levels remain far below the poverty line, the expected effects of a UBI on the reduction of poverty and inequality, empowerment and economic freedom remain an unfulfilled promise’ (Ortiz et al., 2018). However, according to the UBI proposals and experiments presented in a 2018 report, many benefit levels are below the national poverty line (Ortiz et al., 2018). For the TBI, three methods to decide on the payments are presented: ‘top-ups on existing average incomes in each country up to a vulnerability threshold; lump-sum transfers that are sensitive to cross-country differences in the median standard of living; lump-sum transfers that are uniform regardless of the country where people live’ (Molina & Ortiz-Juarez, 2020). The cost of implementing the TBI is based on people earning at least the national poverty line. The UUBI are proposed to be a ‘regular cash transfer that amounts to enough for basic survival’ (Banerjee & Duflo, 2019; Duflo & Banerjee, 2020).

##### Cash transfers increase the incomes and resilience of poor and vulnerable households. The amounts paid may not guarantee a basic or decent standard of living, but large multiplier effects may occur

The available evidence around cash transfers shows that there is sufficient evidence to conclude on a beneficial impact on poverty reduction (Bernstein et al., 2019) but also that there is a great variation in the impacts found (Banerjee et al., 2019; Bastagli et al., 2016). It also appears that in many cases the impact is not big enough to have a direct effect on aggregate poverty levels (Bastagli et al., 2016). Furthermore, with large-scale implementation of money transfers for a longer period of time, the welfare gains depend on where the money comes from to fund the mechanism (Banerjee et al., 2019; Bastagli et al., 2016). One study estimates a large multiplier effect of a long-term cash transfer programme in Kenya (Egger et al., 2019).

##### Possible unintended effects and trade-offs of cash transfers and basic income to be taken into account

There are various unintended negative effects that could materialise when cash transfers or basic income measures are implemented. Such payments should not replace existing social security systems without covering ‘life-cycle contingencies’ which are generally covered by such systems (Ortiz et al., 2018), otherwise the beneficiaries may be worse off in the long run. Additional income may furthermore result in the increase of prices of perishable/protein rich food, which would be a challenge for the poorest households as they spend a large proportion of their income on food (Molina & Ortiz-Juarez, 2020). Such price increases could thus result in food insecurity (Kandpal, 2019), but price inflation may also be minimal (Egger et al., 2019). It also matters whom the payments are made to within a household. If payments are made to individuals regardless of household composition, this avoids ‘within-household discrimination that could be particularly harmful for women’s empowerment and control of economic resources’ (Molina & Ortiz-Juarez, 2020). The ability of a country to afford direct cash payments, particularly when the commodity is a major source of foreign exchange and sold at low prices, has implications for the need to reconsider and model the impacts of national and global commodity market price changes.

#### Pricing policies and supply management to achieve stable and remunerative prices in the long run

##### Supply management could be used to address market failure, and stabilise prices, avoid oversupply and mitigate negative environmental impacts

Stable and remunerative commodity prices are vital to productivity growth, raise farmer incomes and enable the agricultural sector to be an engine of economic development. Commodity price increases work in some niche market segments to increase incomes (Tony’s Chocolonely, 2020) but evidence is lacking that they improve profits for large numbers of farmers at sector level. This raises the discussion about the role of measures that seek to match production with demand. Because higher prices at scale could induce farmers to produce more, they may create a downward pressure on prices (Squicciarini & Swinnen, 2016). One of the solutions for increasing commodity prices without inducing negative effects is to establish a system of internationally agreed supply management to match production with market demand. Production and trade could be managed through buffer stocks, national quotas, measures to limit production to national quotas and to discourage free riding by countries. Such systems could generate funds to pay payments to farmers in a specified area for environmental protection or diversification, taking a conservation and landscape approach to supply management (Koning & Jongeneel, 2008). Such systems avoid the current effects of largely cocoa futures market price is generally used to set national benchmark conventional cocoa prices (Oomes et al., 2016), where there is little opportunity for farmers and processors of conventional cocoa to determine prices, other than in the niche cocoa sector, such as the speciality, fine flavour and some certified chocolate markets (Bonjean & Brun, 2016; Squicciarini & Swinnen, 2016). The international commodity agreements for coffee, cocoa and sugar are examples of such supply management measures, but they collapsed in the 1980s because of opposition from companies and organisations in consuming countries (Koning & Jongeneel, 2008). The International Coffee Agreement (1962–1989) retained export quotas and successfully moderated the coffee price fall until 1989 (Akiyama & Varangis, 1990). In response to the recent cocoa price fall, such a system is explicitly mentioned in the declaration of the 2018 conference of the International Cocoa Organisation (ICCO, 2018). Prerequisites for such systems to work are that there are: relatively few producing countries, no other product that can substitute the commodity, leadership responsibility lying with producing countries, farmers and their organisations are involved in national and macro level pricing decisions, production controls are created in fair and efficient ways, measures to prevent countries and farmers from free riding exist (Koning & Jongeneel, 2008), measures to deal with excess production and how farmers are allocated quotas for remunerative cash crop, such that alternative, income earning opportunities for poor producers are available if farmers are not allowed to expand their production to earn a higher income.

### Interventions addressing food supply system activities

#### Context and market analyses for cost-effectiveness

##### Interventions should include a context and market analysis to ensure effective value chains, without unbalancing supply and demand

The likelihood of achieving impact at scale increases if projects start with a context analysis and a market analysis (Nutz, 2017; UNHCR, 2015). A context analysis includes a socio-economic analysis, providing information on the existing socio-economic situation of the target group and the community they live in. This should include information on their interests, aspirations, risk aversion, financial capabilities, and other aspects that may affect their decision making (Nutz, 2017; UNHCR, 2015). A market and value chain analysis is also necessary to assess the demand for services or products that may be generated through interventions focusing on diversification as well as supply chain logistics. For interventions focusing on productivity enhancement to work, the supply of inputs and access to input markets needs to be guaranteed at the right time with regard to the crop calendar. When the intervention focuses on service delivery, the demand for these services needs to be carefully assessed in addition to demand for the product. For all interventions, it is therefore important to consider the synergy between supply and demand, locally but if implemented at scale, also regionally or even globally.

##### To facilitate farmers’ behavioural change, interventions need to address contextual and personal factors

The contextual factors shown in Fig. 3 can affect how increased income from agricultural production and off-farm activities interact. Interventions that address these barriers are therefore more likely to lead to behavioural change of farmers and therefore positively impact incomes in a cost effective way. In the interventions we studied, the focus was limited to community and farm level inputs, extension and financial services and in some cases, support to market produce, education and environmental protection. Most interventions focus on commodity income and some on diversification, while another focus may more relevant for farmer and his/her households. The same holds for the importance of addressing personal factors**,** as they also influence farmer and household decision-making processes. In the interventions studied, personal factors were not explicit in the intervention design. Taking farmers’ and household’s personal factors explicitly into account in design of interventions will enhance their effectiveness and efficiency.

#### Price increases by companies

One of the activities that supply chain companies such as traders, manufacturers and retail can do to increase incomes is to increase the farmgate price paid to farmers. As so many commodity farmers are poor, every price – and thus income- increase is worthwhile. Information about the potential for such price increases to achieve living incomes can be found in Sect. 3.3.1. In that section we conclude that for large groups of vulnerable farmers, high price increases will, even though they will increase incomes, not lead to such farmers earning a living income. The poorest farmers benefit least because of selling small volumes. Advantages and disadvantages of increasing prices can further be found in Sect. 4.1.5. Finally, a question is whether companies have enough financial room to increase payments significantly to the sometimes millions of farmers they source from, for instance by establishing a relatively high minimum farmgate price, decreasing price volatility. There is, however, an evidence gap with regard to such information.

### Interventions addressing environmental drivers

#### Agro-ecological and climatic conditions should be favourable for farmers to achieve a living income

##### Environmental drivers are key for thriving food systems, but long and short term interests are conflicting

Many commodity producers in lower- and middle-income countries are expected to experience the impacts of changes in climate (Centro Internacional de Agricultura Tropical, 2011; Läderach et al., 2013; Masters et al., 2010; Ovalle-Rivera et al., 2015). To achieve sustainable changes in farmer incomes, a long-term perspective is needed. If according to climatic predictions it will no longer be possible to produce key commodities in a certain region in the future, these farmers must be guided in shifting their production to other activities or in earning income from off-farm sources. There are also other trade-offs between short- and long-term interests. An important example concerns deforestation. In cocoa farming, for example, much forested land has been converted to cocoa plantations in the past. The traditional shade management has been gradually replaced by full-sun monoculture (Franzen & Mulder, 2007). Such full-sun, monocrop cocoa systems enhance yields in the short term but these yields may not be maintained in the long run. Farmers who are unaware of this, and farmers with a short time horizon can be inclined to opt for such a farming system, which leads to severe long-term soil nutrient degradation, in turn leading to very low levels of productivity in the long run, as the pressure on the land is too high to leave it fallow after 20 to 30 years of intensive use (Ruf, 2001).

## Conclusions and recommendations

In this chapter we conclude on how different types of smallholder commodity farmers can be supported to achieve a living income and give recommendations for policymakers of public and private sector organisations for designing and implementing interventions to achieve this goal.

### A large proportion of smallholder cocoa and tea households do not have the potential to earn a living income without structural change

Despite huge investments in improving the livelihoods of smallholder tree-crop commodity farmers in the past decades, there is still widespread poverty amongst coffee, cocoa, tea, and palm oil producers. The impact of interventions, if any, has not been sufficient for farmers to be lifted out of poverty at scale, nor has resulted in large groups of farmers earning a living income. Interventions that only address a single food system component (for instance productivity or price increases) have not lifted the majority of farmers above the extreme poverty benchmark. The reason for the lack of success is that in many cases the underlying poverty drivers were not addressed, such as land fragmentation, volatile prices, buying prices which do not cover all costs, farmers’ lack of capacity to invest and difficulties for farmers to diversify income sources on-farm and off-farm (such as employment). A large proportion of cocoa and tea farmers for whom we have collected data therefore do not have the potential to earn a living income.

### A starting point for short- to medium-term interventions for households without the potential to earn a living income should be to improve their resilience

For farmers who do not have the possibility to improve their income, support could focus on a one health approach which embraces food security and nutrition (Walton et al., 2020, Arsyad et al., 2019), alternative on- and off-farm income and employment, and social assistance programmes. For those farmers it often does not make sense to focus efforts on commodity production. Therefore, depending on their circumstances, they can best be supported in finding employment, and improving their resilience through improving food production for home consumption, water, sanitation, hygiene as well as access to healthcare and education possibilities. Social assistance programmes such as cash transfers or basic income schemes should also be considered. This protects them against the likelihood of falling back to poverty if unexpected circumstances occur.

### Long term policies such on land governance, labour market development and social assistance should be considered, but even they do not necessarily guarantee a living income to be achieved

If small farm sizes hamper many farmers in earning a decent income, rights-based land governance policies could be developed with participation from the concerned communities. Such policies need to take into account expected climate change effects as in many tree-crop commodity sectors it is expected that climatic conditions in major production zones will change in the future. Land policies should take into account forest and biodiversity protection and fragmentation on a landscape level and anticipate indirect, tele-coupled impacts from interventions. Together with facilitating household members to earn an income through employment (either within agriculture or other sectors), this could create opportunities for some farmers to increase their farm and improve incomes and for others to move out of self-employed agriculture in this generation or the next. This is obviously not easily achieved as different stakeholders often have different priorities, and employment opportunities are not easily created and if so, people do not tend to easily move away from their community and social network. Also, being employed does not guarantee earning a living wage because minimum and prevailing wages are generally lower than a living wage. In addition, cash transfers and a Universal Basic Income are expected to increase incomes, but also do not guarantee earning a living income directly though indirect welfare effects could occur.

### There is no silver bullet to achieve a living income for all smallholder tree-crop commodity farmers because different groups of farmers need different types of support. The private sector, governments and NGOs all have a role to play

As different types of farmers have different types of needs, there is no silver bullet to achieve a living income for all smallholder tree-crop commodity producers. Interventions by the private sector and NGOs can support many farmers achieving living incomes and support farmers and communities to increase resilience. But they generally cannot achieve living incomes for the most vulnerable farmers as many of the structural factors hampering income increases for such farmers are beyond their sphere of direct influence. Governments have an important role in addressing structural factors and, together with market parties, have a role in transforming the markets and thus prices locally, nationally and internationally. A shift towards more system-oriented strategies in multi-stakeholder setting appears most likely to benefit the scale and sustainability of impact.

### Achieving living incomes requires talking to farmers and their household members, coordination between stakeholders, sharing lessons learnt and data

In assessing the potential for households to earn a living income from commodities the focus should be on whether farmers can reasonably be expected to reach a decent standard of living. It is important to talk to farmers about their needs, wants and aspirations, and take contextual and personal factors into account in designing interventions, within households and within communities. The effective implementation of diversified interventions requires coordination between all stakeholders active in a given region, landscape and/or value chain. Each stakeholder has their own strengths to contribute: buying companies can support those farmers for whom it is relevant with agronomic advice and input services, and work alongside other stakeholders (such as other supply chain actors for diversification) to improve the situation of their supplying farmer households who cannot improve commodity income. Improving land governance, health care, education, water, sanitation and food security is generally the role of governments, as are social assistance programmes. Interventions focusing on price and supply management are generally the domain of both governments and private sector. To assess whether households have the potential for earning a living income based on a specific commodity cash crop, there is a need for data sharing to limit the burden of farmers being asked the same questions multiple times. Data disaggregated by especially gender, but also age and other socio-demographic characteristics can aid to close the current data gap.

## Data Availability

UTZ (now Rainforest Alliance) consented to use data on behalf of all the funders of the cocoa studies.

## Appendix 1 Information from the literature review on the impact of interventions on crop and household income

### Contract farming

The systematic review finds that the effects are positive (RR = 1.32 on household income – 8 studies, RR = 1.65 on farming income – 6 studies, RR = 1.92 on contracted crop income – 12 studies). However, there seems to be a very large selection and survivor bias, with a high likelihood of an overestimation of effects. However, the authors note that it is likely that the effects will be significant regardless, as smallholders would not give up their farming autonomy (or maintain doing so over the years) if they would not be (Ton et al., 2017).

### Standards and certification

The systematic review finds the effects on the total household income of farmers are unclear (= coffee (3), cocoa, banana, horticulture, black pepper, other; 8 studies in total). While household incomes of farmers engaged in certified production were 6% higher than those of households not engaged in certified production, the overall effect is not statistically significant (SMD = 0.13). The effect size estimated for individual studies range from negative to positive, though all statistically significant studies provided positive estimates. Effects on crop income (= coffee (4), horticulture (2), cocoa (2), tea, other) were positive and statistically significant (SMD = 0.22). Incomes from the sale of produce were 11% higher if the produce was certified (Oya et al., 2017). The Farmer Income Lab (3 sources assessed in detail) identifies this as medium income impact (10–50% income increases) with demonstrated limited impact on income enabling factors (high scale, medium durability) (Dalberg & Wageningen University, 2018).

### Cash transfers

The systematic review examined nine studies with impacts on poverty measures (poverty headcount, poverty gap), out of which about 2/3rds find a significant impact. While cash transfers were shown to lead to an increase of total and food expenditure for most programmes, it appears that in many cases this impact is not big enough to have an effect on aggregate poverty levels. Findings range from about 4 percentage points to 8 or 9 percentage points, depending on the measure of poverty. But, the long term effects are not clear yet (Bastagli et al., 2016).

### Agricultural input subsidies

The systematic review finds that the effect of receiving fertiliser and seed subsidies on income and household expenditures, on average, is 0.15 (significant). All studies included (3 sources in total) report a positive impact, but there is a high degree of between-study variability, which is most likely caused by the different outcome variables between the studies. The effect sizes for income and expenditures are smaller than those for revenue. Two studies on seed inputs for maize crops measured the effects on poverty, one found an 11 percent decrease in the numbers of farmers living beneath the USD 1.25 poverty line and a smaller 7 per cent decrease in those living beneath the USD 2.00 poverty line, whereas the other found no significant effect on the severity of farm household poverty (the degree of inequality below the poverty line). The meta-regressions also show small, negative relationships between subsidy size and yield as well as between subsidy size and income. However, these relationships are not statistically significant. Consequently, the meta-regression analysis provides no evidence of an association (positive or negative) between subsidy size and agricultural outcomes (Hemming et al., 2018). The Farmer Income Lab identifies input subsidies (24 sources assessed in detail) as an intervention that did not show significant income increases (< 10%). The scale was identified as high, durability as low (Dalberg & Wageningen University, 2018).

### Access to finance

The Farmer Income Lab (Dalberg & Wageningen University, 2018) (13 sources assessed in detail) identified this as medium income impact at scale with demonstrated impact on income enabling factors. Interventions demonstrated income increases of 15% on average and the ability to reach between 2,100 and 400,000 clients. These programs are effective because they: 1) segmented farmers, 2) tailored solutions to farmers, 3) leveraged farmer aggregation, and 4) bundled services.

### Productivity enhancement through training & input services

Many projects focus have a primary focus on increasing farmer productivity, as it is thought to benefit both the buying companies as well as the farmers. The assumption behind such projects is that farmer productivity leads to increases in farmer profits and/or household incomes, however the effect of such projects on household income is not always as clear as expected. The adoption of good agricultural practices, including sustainable agricultural practices, is frequently associated with increases in (household) income (see e.g.: Ali & Abdulai, 2010; Amare et al., 2012; Asfaw et al., 2016, 2019; Kassie et al., 2018; Noltze et al., 2013; Pray & Huang, 2003; Teklewold et al., 2013). However, the question remains whether training and/or input services lead to actual changes in the adoption of practices and, if yes, for how long. Moreover, the adoption of good agricultural practices is also often associated with increases in costs of production, which is also yields some studies that find no changes in household income (Takahashi & Barrett, 2014) or profits (Takahashi et al., 2019) due to these increased costs, or even negative effects on income (Daniel et al., 2010). Moreover, if all farmers would have increases in yields, the prices might decrease in the long run, eliminating any income effects for farmers. The Farmer Income Lab classifies productivity enhancement as a mixed evidence of impact, demonstrating between 10–50% income improvements, with medium reach and durability (Dalberg & Wageningen University, 2018).

### Farmer field schools

The most common strategies for productivity enhancement are providing training in agricultural practices and providing input services, which are also frequently combined in projects. The approaches to reaching farmers can differ. Many projects provide their services directly via cooperatives, while others use a more bottom up approach and use Farmer Field Schools (FFSs) to disseminate knowledge. A systematic review on FFSs found that a significant increase in agricultural yields was found, by 13 per cent over comparison farmers (RR = 1.13, 11 studies). They also found a significant increase in profits (net revenues), by 19 per cent among FFS participants over comparison farmers (RR = 1.19, 2 studies). They mention that the increase in profits was higher for FFS projects which also included complementary interventions involving input or marketing support (RR = 2.51, 2 studies) (Waddington et al., 2014). It is important to note here that commodity profits do not directly translate to significant increases in total household income, which is not something that was included in the studies reviewed. The Farmer Income Lab identifies FFSs as an intervention with mixed evidence of impact, as it demonstrates between 10–50% income improvements, with high reach but low durability (Dalberg & Wageningen University, 2018).

## Appendix 2 Calculating poverty lines and living income benchmarks to compare data between countries

### Living income benchmark calculations

For each country, household incomes were converted to match the living income benchmark:

- Ghana: (Smith & Sarpong, 2018)
- Cocoa Côte d’Ivoire: (Tyszler et al., 2018)
- Kenya: (Anker & Anker, 2015)

The monthly living income benchmarks were converted to the year of each dataset using the changes over time in the consumer price index. The living income benchmark is based upon a country specific average family size (6 in Côte d’Ivoire, 5 in Ghana, 5.5 in Kenya). Therefore, yearly household income from each of the datasets was adjusted only for the period: it was divided by 12 to change the data from yearly to monthly income. For comparison with the World Bank poverty line, the monthly living income line per family was converted to a daily living income per household member. By doing so, we treated adults and children in the households in the same way, not correcting for male or female equivalent FTE values. We did not deduct from the benchmark figures the value of food produced for home consumption, as such information was not available. Therefore, we assume all farmers buy all food. As farmers likely produce some food themselves, this assumption creates an overestimation of the living income gap in our calculations.

Finally, the living income benchmark was adjusted to the year of the surveys using changes in the consumer price indices. After correction, the living income benchmark is equivalent to CFA 1,450 per person per day for Côte d’Ivoire in 2017, to 5.747 Cedi per person per day in Ghana in 2014 and to 135.94 Kenyan Shillings per person per day in Kenya in 2015.

### Poverty line benchmark calculations

For each country, household incomes were converted to match the poverty line of USD 1.90 per person per day (2011 PPP). This poverty line was set in 2011, and was adjusted to the year of the data and local currencies using the PPP conversion factor of 2011 and the changes in time using the consumer price index, following the methodology as used by USAID (Sillers, 2005). After correction, the USD 1.90 poverty line is equivalent to CFA 480 in 2017 for Côte d’Ivoire, to 2.068 Cedi in 2014 for Ghana, and to 73.63 Kenyan Shillings in 2015 for Kenya. The yearly household level income data was converted to daily income by dividing by 365, and then divided by the number of household members.

## Appendix 3 Calculations on the effect of price increases on income

The ICCO daily price was USD 2501/tonne on 18 September 2020 (ICCO, 2020). The Living Income Differential by the governments of Ivory Coast and Ghana in 2019 translates to USD 400/tonne on all 2020/2021 season cocoa contracts, on top of the market price (Angel et al., 2019; Vidzraku, 2018). This is a 16% increase compared to a market price of USD 2501/Mt. The mean income of farmers earning under the living income benchmark as well as under the World Bank extreme poverty line (USD 1.90 PPP) in Ghana is USD 0.27 per person per day, out of which 84% stems from cocoa farming (Waarts et al., 2015). A 16% increase on 84% of USD 0.27 generates a new income of USD 0.307 per person per day, which is an increase of USD 13.3 per person per year compared to a situation without the Living Income Differential.

The minimum Sustainability Differential as announced by Rainforest Alliance is USD 70/Mt (Rainforest Alliance, n.d.), which is a 3% increase compared to a market price of USD 2501/Mt. A 3% increase on 84% of USD 0.27 generates a new income of USD 0.277 per person per day, which is an increase of USD 2.5 per person per year compared to a situation without the Living Income Differential.

## Footnotes

1 In the actual net income measurement, food produced by the household and used for home-consumption is taken into account, as many smallholder farmers consume food produced by themselves.

2 The following benchmarks were used for the living income comparisons: (Anker & Anker, 2015; CIRES, 2018; Smith & Sarpong, 2018).

3 Findings range from about 4 percentage points to 8 or 9 percentage points increase total and food expenditure, depending on the measure of poverty.

4 The population of Sub-Saharan Africa, for example, is expected to double by 2050. Source: https://blogs.worldbank.org/opendata/worlds-population-will-continue-grow-and-will-reach-nearly-10-billion-2050

5 We consider factor 6, the number of household members out of scope for interventions but it is an important factor to be taken into account as it affects the money available per household member.

6 We do not include an analysis on how to address the number of household members, as we see that as beyond the scope of action. However, it remains an important factor to take into account as the number of household members has a significant influence on the capability of a household to earn a living income. The reason for this is that to earn a living income, the living income benchmark needs to be met regarding household income per household member per day. With large households, a high income needs to be earned to meet the living income benchmark (see also Sect. 1.2).

7 In addition, there are cases of common land or open access tenure where there is no control on access to resources.

8 It is dangerous to generalise about African land tenure systems, as they are diverse, dynamic, shaped by laws, policies, contexts (Atwood, 1990).

9 This is reflective of the prevalence of dual legal systems: as statutory land laws were introduced by colonial authorities, local communities often continued to hold land under ‘traditional’ customary systems. But even in states that were not formally colonised or occupied, land formalisation has taken place well before World War II (Putzel et al., 2015).

10 Universal Basic Income (UBI) or Universal Ultra Basic Income (UUBI).

11 They assume the National poverty line ensures such a basic standard of living.

## Code availability

All calculations and analyses for graphs were conducted using Stata 14. Code is available on request.
